# Banana Passion Fruit-Mediated Green Synthesis of Copper(I) Iodide Nanoparticles for Concrete Biodeterioration Control: Antimicrobial Activity, Cytotoxicity, and Mechanical Compatibility

**DOI:** 10.3390/nano16160976

**Published:** 2026-08-08

**Authors:** Samantha Fajardo, Andrés Izquierdo, Ana G. Haro-Báez, Alexis Debut, Geovanna Arroyo, Andrea Aluisa, Marbel Torres Arias, Hugo Bonifaz, Juan Haro, Carlos Navas-Cárdenas, Erika Murgueitio Herrera

**Affiliations:** 1 Departamento de Ciencias de la Vida y la Agricultura, Universidad de las Fuerzas Armadas ESPE, Av. Gral. Rumiñahui s/n, Sangolquí P.O. Box 171-5-231B, Ecuador; smfajardo@espe.edu.ec (S.F.); arizquierdo@espe.edu.ec (A.I.); acaluisa@espe.edu.ec (A.A.); mmtorres@espe.edu.ec (M.T.A.); 2 Centro de Nanociencia y Nanotecnología, Universidad de las Fuerzas Armadas ESPE, Av. Gral. Rumiñahui s/n, Sangolquí P.O. Box 171-5-231B, Ecuador; apdebut@espe.edu.ec (A.D.); gvarroyo@espe.edu.ec (G.A.); 3 Departamento de Ciencias de la Tierra y de la Construcción, Universidad de las Fuerzas Armadas ESPE, Av. Gral. Rumiñahui s/n, Sangolquí P.O. Box 171-5-231B, Ecuador; agharo@espe.edu.ec (A.G.H.-B.); hfbonifaz@espe.edu.ec (H.B.); jfharo@espe.edu.ec (J.H.); 4 Departamento de Ciencias de la Energía y Mecánica, Universidad de las Fuerzas Armadas ESPE, Av. Gral. Rumiñahui s/n, Sangolquí 171103, Ecuador; canavas3@espe.edu.ec

**Keywords:** copper(I) iodide nanoparticles, green synthesis, taxo extract

## Abstract

This study aimed to synthesize copper(I) iodide nanoparticles (CuI NPs) through a green route using taxo (banana passion fruit) extract as a natural capping and stabilizing agent, and to evaluate their antimicrobial performance against microorganisms isolated from concrete, together with a preliminary cytotoxicity screening. The obtained nanoparticles were characterized by ultraviolet–visible spectroscopy (UV–Vis), Fourier-transform infrared spectroscopy (FTIR), transmission electron microscopy (TEM), scanning electron microscopy coupled with energy-dispersive X-ray spectroscopy (SEM–EDS), dynamic light scattering (DLS), and X-ray diffraction (XRD). UV–Vis spectra recorded in the 200–704 nm range showed a strong absorption band at 224 nm, consistent with electronic transitions associated with nanostructured CuI. FTIR analysis revealed extract-derived biomolecules adsorbed on the nanoparticle surface, with bands assigned to aliphatic C–H, aromatic moieties, and C–O/C–O–C vibrations, supporting the formation of an organic capping layer. DLS analysis showed a mean hydrodynamic diameter of approximately 32 nm in aqueous suspension, whereas TEM revealed particle sizes ranging from 13 to 42 nm. XRD confirmed a predominantly cubic CuI phase, while SEM–EDS identified Cu and I as the main elements, with minor signals attributed to residual organic coating and/or trace species from the synthesis medium. The CuI NPs exhibited antimicrobial activity against microorganisms isolated from medium-strength concrete, producing inhibitory effects at all tested concentrations (0.014, 0.0087, and 0.0035 mol/L). Preliminary cytotoxicity screening using the 3-(4,5-dimethylthiazol-2-yl)-2,5-diphenyltetrazolium bromide (MTT) assay in human foreskin fibroblast (HFF), human breast adenocarcinoma (MCF7), and human glioblastoma (U251) cell lines showed dose- and time-dependent reductions in metabolic viability. The 1.0 mol/L formulations, particularly the precipitated fraction, produced stronger cytotoxic effects, whereas the 0.1 mol/L formulations, especially the residual fraction, preserved comparatively higher metabolic viability. Overall, these findings suggest that taxo-mediated CuI NPs are promising antimicrobial candidates for concrete biodeterioration control, while further colloidal and biological studies are required to better define their behavior under cell-culture conditions and optimize their safe application.

## 1. Introduction

Microbial deterioration of construction materials, particularly concrete, is a global challenge associated with high maintenance and restoration costs [1]. The colonization of mineral surfaces by bacteria, fungi, and algae promotes biofilm formation, while microbial metabolism progressively compromises the structural and chemical integrity of the substrate [2,3]. This biodeterioration process may lead to cracking, loss of cohesion, and increased porosity, particularly under conditions of high humidity, solar radiation, and thermal fluctuations [1,4,5]. Conventional control strategies have relied mainly on mechanical removal and the application of broad-spectrum chemical biocides. However, these approaches often provide only temporary protection and raise concerns related to environmental toxicity, human health risks, and the development of microbial resistance [1,5,6,7]. In particular, halogenated and organomercurial synthetic biocides, despite their effectiveness, are increasingly restricted by international regulations due to their ecotoxicity [1,7]. These limitations have encouraged the development of more sustainable alternatives based on eco-friendly materials and green-derived nanocomposites [8].

Metal-based nanoparticles have emerged as promising antimicrobial agents because of their high surface reactivity and broad-spectrum activity [9,10]. Among them, copper-based nanomaterials are particularly relevant due to their ability to damage microbial cells through multiple mechanisms, including reactive oxygen species (ROS) generation, disruption of membrane integrity, and denaturation of essential biomolecules [11,12,13,14]. Iodine (I_2_), in turn, is widely used as an antiseptic in hospital and food-disinfection settings because of its well-established antimicrobial performance and safety profile [15,16]. Therefore, the combination of copper and iodine in copper(I) iodide nanoparticles (CuI NPs) may provide enhanced antimicrobial activity through contact-mediated effects and controlled Cu+/I− release [14,17]. Although CuI-based systems have been mainly explored in textiles and polymeric materials, their antimicrobial mechanisms may also be useful for mineral coatings or additives intended to protect construction materials. In addition, immobilization within mineral or polymer matrices may help reduce leaching while maintaining surface-mediated antimicrobial activity [18,19,20,21,22].

Green synthesis using plant extracts offers an environmentally friendly route for producing metal-based nanoparticles. Its scientific value depends not only on replacing conventional reagents, but also on the biological source, the metabolites or biopolymers involved, the nanoparticle system obtained, and the intended application. Plant-derived compounds and biopolymers such as polyphenols, flavonoids, anthocyanins, tannins, organic acids, polysaccharides, proteins, lignin, and other oxygenated biomolecules can participate in metal-ion coordination, nucleation, growth control, surface capping, and colloidal stabilization [23]. In particular, lignin is a structurally defined aromatic biopolymer rich in phenolic and oxygenated functional groups, which makes it a relevant plant-derived system in green nanoparticle synthesis [24]. These functional groups may contribute to metal-ion binding, nanoparticle stabilization, and the formation of an organic surface layer that influences nanoparticle reactivity and biological performance. Consequently, green synthesis may provide an organic surface environment capable of modulating nanoparticle stability, reactivity, and biological activity. In this context, underexplored Andean biomass sources represent a valuable opportunity to develop sustainable nanomaterials for locally relevant applications [25,26].

Taxo, or banana passion fruit (*Passiflora tripartita*), is an Andean fruit containing polyphenols and other functional compounds that may contribute to antioxidant and biological activity [26,27]. Flavonoids and anthocyanin pigments reported in *Passiflora* species have also been associated with antioxidant capacity and antimicrobial effects [26,28,29]. However, compared with other species such as passion fruit or maracuya (*Passiflora edulis*), taxo remains less studied in terms of its physicochemical properties, applications, and potential use in green nanotechnology. Previous studies on *P. edulis* have reported the presence of flavonoids, tannins, polyphenols, and bioactive peptides with antibacterial and antifungal activity, including inhibitory effects against *Bacillus subtilis*, *Staphylococcus aureus*, *Trichoderma harzianum*, *Fusarium oxysporum*, and *Aspergillus fumigatus* [28,30,31]. These findings support the potential of *Passiflora*-derived metabolites as functional components in nanoparticle synthesis.

In this study, taxo extract was used as a natural reducing, stabilizing, and capping agent for the green synthesis of CuI NPs. The novelty of this work lies in the use of an underexplored Andean biomass source for CuI nanoparticle formation and in the evaluation of the resulting material as an antimicrobial agent for concrete biodeterioration control. Although plant-mediated nanoparticle synthesis has been widely reported [23], limited information is available on the use of *Passiflora tripartita* metabolites for CuI NP synthesis, their interaction with microorganisms isolated from concrete, and their compatibility with cementitious materials. This approach therefore differs from conventional green-synthesis studies focused only on nanoparticle preparation or general antimicrobial screening.

The cytotoxicity of CuI NPs must also be considered for their safe application. Previous cell-culture studies have reported dose-dependent cytotoxic effects associated with oxidative stress, reduced viability, and apoptosis-related pathways [32,33,34]. Evidence from copper and copper oxide nanoparticles further indicates that Cu^+^/Cu^2+^ release, intracellular dissolution, ROS generation, membrane disruption, and mitochondrial dysfunction may contribute to cytotoxic and genotoxic responses [11,34,35,36]. Although these mechanisms are plausible for CuI, its toxicological behavior may differ from that of CuO or Cu^0^ because of the chemistry of I^−^ and the stability of the γ-CuI phase [37,38,39,40]. In ecotoxicological contexts, specific information on CuI remains limited; however, copper-based nanoparticles are generally known to produce concentration- and size-dependent effects in aquatic organisms, including oxidative stress and developmental alterations [41,42]. These findings support the need for cautious formulation, discharge management, and standardized ion-release assessment.

Accordingly, the present work aimed to synthesize CuI NPs using taxo extract through a green route and to evaluate their potential as antimicrobial additives or coatings for medium-strength concrete. The proposed strategy seeks to reduce microbial colonization and biofilm formation through contact-mediated inactivation and controlled Cu^+^/I^−^ release, while minimizing leaching by immobilization within the mineral matrix. To assess applicability, antimicrobial activity was evaluated against microorganisms isolated from concrete, and biocompatibility was examined using the 3-(4,5-dimethylthiazol-2-yl)-2,5-diphenyltetrazolium bromide (MTT) assay to support dosage optimization and safer performance in cementitious materials.

## 2. Materials and Methods

### 2.1. Materials

Fresh banana passion fruits were purchased from a Supermaxi supermarket in Sangolquí, Pichincha, Ecuador. Copper(II) sulfate pentahydrate (CuSO_4_·5H_2_O; Certified ACS; CAS No. 7758-99-8; Fisher Scientific Company, Fair Lawn, NJ, USA) and potassium iodide (KI; Fisher Scientific Company, Fair Lawn, NJ, USA) were used for nanoparticle synthesis. Double-deionized water was produced using a Thermo Scientific Barnstead Smart2Pure water purification system (Thermo Electron LED GmbH, Langenselbold, Germany).

### 2.2. Preparation of Banana Passion Fruit Extract, Taxo

Banana passion fruit extract was prepared following the protocol reported in [25], with minor modifications. Briefly, the fruits were thoroughly washed with mild detergent and Type I water, air-dried at room temperature, and peeled. The edible fruit material was subjected to hydroethanolic extraction using 70% ethanol at an ethanol-to-fruit ratio of 2:1, under orbital agitation for 4 h. The obtained extract was filtered several times through a 250 µm filter to remove suspended solids and ensure sample homogeneity. The filtered extract was stored at 4 °C until use. Ultrapure water from a Milli-Q system (conductivity: 0.28 µS/cm; Millipore Direct-Q, Merck KGaA, Darmstadt, Germany) was used for the preparation of reaction solutions and purification of the synthesized products.

### 2.3. Characterization of Fruit|

The antioxidant activity of the banana passion fruit extract was evaluated using the 2,2-diphenyl-1-picrylhydrazyl (DPPH) assay with a Genesys 10 UV–Vis spectrophotometer equipped with VISIONlite Scan software, version 4.0 (Thermo Fisher Scientific, Waltham, MA, USA). All measurements were performed in triplicate according to the manufacturer’s instructions. The experimental data were analyzed using Minitab Statistical Software (version 21.3; Minitab LLC, State College, PA, USA). Significant differences among mean values were assessed by multifactorial analysis of variance (ANOVA), followed by Fisher’s least significant difference (LSD) test at a significance level of *p* < 0.05.

For Fourier-transform infrared spectroscopy (FTIR) analysis, the banana passion fruit extract obtained after ethanolic extraction, centrifugation, and lyophilization was characterized using a PerkinElmer Spectrum 3 spectrometer (PerkinElmer Inc., Shelton, CT, USA). Attenuated total reflectance Fourier-transform infrared spectroscopy (ATR-FTIR) spectra were recorded in absorbance mode at a spectral resolution of 4 cm^−1^, using Happ–Genzel apodization, a KBr beam splitter, and a DLATGS detector. Each spectrum was acquired by averaging 50 scans. After baseline correction, the spectra were processed and analyzed using SpectraGryph software (version 1.2.16.1; Dr. Friedrich Menges, Oberstdorf, Germany), including visualization, normalization, and assignment of characteristic absorption bands [43].

### 2.4. Synthesis of CuI Nanoparticles

CuI nanoparticles were synthesized following the protocols reported in [37,39,44], with modifications. Briefly, a 0.1 mol/L CuSO_4_ precursor solution was prepared using copper sulfate pentahydrate (CuSO_4_·5H_2_O), while a 0.2 mol/L KI solution was prepared separately. Equal volumes of both precursor solutions were mixed at a 1:1 *v*/*v* ratio under magnetic stirring at room temperature. Subsequently, the previously filtered banana passion fruit extract was added to the precursor mixture at a precursor mixture-to-extract ratio of 10:1 *v*/*v*. This ratio was selected to provide sufficient phytochemical content for surface stabilization and capping, while maintaining the reaction conditions required for CuI precipitation. The reaction mixture was kept under magnetic stirring until a visible color change and precipitate formation were observed.

The resulting precipitate was thoroughly washed with Milli-Q water to remove unreacted ions and residual soluble species. The suspension was then centrifuged using a Hermle Z206A centrifuge (HERMLE Labortechnik GmbH, Wehingen, Germany) and sequentially filtered through filter paper, followed by 0.45 μm and 0.22 μm membrane filters. The final products were stored in airtight bottles at room temperature and protected from light until characterization.

To evaluate synthesis reproducibility, all preparations were carried out under the same precursor concentrations, extract ratio, stirring conditions, washing procedure, and storage conditions. Reproducibility was supported by the consistent formation of the characteristic CuI precipitate and by complementary characterization using UV–Vis spectroscopy (Genesys 10uv; Thermo Fisher Scientific, Waltham, MA, USA), X-ray diffraction (XRD) (Empyrean; Malvern Panalytical B.V., Almelo, The Netherlands), transmission electron microscopy (TEM) (Tecnai G2 Spirit Twin; FEI Europe B.V., Eindhoven, The Netherlands), dynamic light scattering (DLS) (LB550; HORIBA Ltd., Kyoto, Japan), and scanning electron microscopy coupled with energy-dispersive X-ray spectroscopy (SEM–EDS) (MIRA 3, TESCAN ORSAY HOLDING, a.s., Brno, Czech Republic; XFlash^®^ 6|30, Bruker Nano GmbH, Berlin, Germany). The concentrations reported for the antimicrobial and cytotoxicity assays should be considered nominal CuI-equivalent concentrations, calculated from precursor stoichiometry and subsequent dilution of the nanoparticle suspensions.

The actual post-synthesis nanoparticle concentration, particle number concentration, and organic-to-inorganic mass ratio were not directly quantified in this study. Therefore, the reported concentrations do not represent exact measurements of purified nanoparticle mass or particle number. Future work should include gravimetric dry-mass analysis, inductively coupled plasma optical emission spectroscopy (ICP-OES), inductively coupled plasma mass spectrometry (ICP-MS), thermogravimetric analysis (TGA), X-ray photoelectron spectroscopy (XPS), and/or carbon, hydrogen, nitrogen, and sulfur elemental analysis (CHNS) to quantify the inorganic fraction, organic coating contribution, and actual nanoparticle concentration.

### 2.5. Characterization of Nanoparticles

UV–Vis analysis was performed using a Genesys 10uv UV–Vis spectrophotometer equipped with Thermo Scientific VISIONlite Scan software, version 4.0 (Thermo Fisher Scientific, Waltham, MA, USA). The instrument undergoes annual maintenance, and analyses were carried out according to the equipment manual. Deionized water was used as the reference for baseline correction. Automatic self-calibration was performed prior to analysis to ensure adequate wavelength accuracy and measurement reproducibility.

The hydrodynamic size distribution of the nanoparticles was determined by dynamic light scattering (DLS) using a HORIBA LB550 particle size analyzer (HORIBA Ltd., Kyoto, Japan), following the manufacturer’s user manual [45].

The crystalline structure of the CuI nanoparticles was analyzed by X-ray diffraction (XRD) using an Empyrean diffractometer (Malvern Panalytical B.V., Almelo, The Netherlands) equipped with a copper X-ray tube (Empyrean Cu LFF) and Cu Kα radiation. The diffraction patterns were recorded over a 2θ range of 5–90°, operating at 45 kV and 40 mA.

The morphology and primary particle size of the nanoparticles were evaluated by transmission electron microscopy (TEM). For sample preparation, a drop of the colloidal nanoparticle suspension was deposited onto a carbon-coated copper grid and allowed to dry by solvent evaporation. The grid was then analyzed using a FEI Tecnai G2 Spirit Twin transmission electron microscope (FEI Europe B.V., Eindhoven, The Netherlands) operating at 80 kV. Instrument calibration was verified using a gold nanoparticle standard, considering the characteristic 0.24 nm spacing. The calibration reference used is available at https://www.agarscientific.com/tem/calibration-standards (accessed on 1 April 2020). Nanoparticle size analysis was performed using ImageJ-win64 software (ImageJ version 1.54p; National Institutes of Health, Bethesda, MD, USA) [46].

Semi-quantitative elemental composition was determined by scanning electron microscopy coupled with energy-dispersive X-ray spectroscopy (SEM–EDS), using a TESCAN MIRA 3 SEM microscope (Brno, Czech Republic) equipped with a Bruker XFlash^®^ 6|30 SDD EDS detector (Berlin, Germany; energy resolution of 123 eV at Mn Kα) [47,48]. Annual maintenance, alignment verification, and calibration were performed following standard quality-control procedures and calibration guidelines [49,50].

### 2.6. Collection and Selection of Microorganisms from Concrete

Three sampling campaigns were conducted on medium-strength concrete with a specified compressive strength of 240 kg/cm^2^. This strength was selected because it is commonly used in Ecuadorian construction and therefore provides a representative scenario for evaluating microbial colonization and activity on cementitious materials. A total of 28 colonies were isolated from the collected samples. Based on macroscopic morphology, relative density, and frequency of occurrence, five predominant microorganisms were selected for further analysis, including two bacterial isolates and three fungal isolates.

Sampling was performed using sterile swabs. The concrete surface was rubbed with the swab, which was then immersed in sterile physiological saline solution prepared with 0.85 g of NaCl (Merck KGaA, Darmstadt, Germany) in 100 mL of distilled water to preserve microbial viability until isolation [51,52]. Serial dilutions of 1:10, 1:100, and 1:1000 were prepared, and 0.1 mL aliquots of each dilution were inoculated onto Petri dishes containing Nutrient Agar for bacterial growth and Rose Bengal Agar supplemented with chloramphenicol (M640; HiMedia Laboratories Pvt. Ltd., Thane, Maharashtra, India) for fungal growth. In parallel, sterile swabs previously moistened with physiological saline solution were rubbed directly over the concrete surface and streaked onto Petri dishes containing Potato Dextrose Agar (PDA) and Rose Bengal Agar. The plates were incubated at 28 °C until visible microbial growth was observed.

The most prevalent isolates were selected from the three sampling campaigns based on their frequency, relative density, and repeatability of macroscopic characteristics, including colony morphology, color, and growth pattern. Microscopic characterization was performed using Gram staining (Merck KGaA, Darmstadt, Germany) for bacteria and lactophenol cotton blue staining for filamentous fungi. After selection, pure fungal cultures were obtained on PDA (BD Difco™, REF 213400; Becton, Dickinson and Company, Sparks, MD, USA), while bacterial isolates were purified and maintained on Nutrient Agar (BD Difco™, REF 213000; Becton, Dickinson and Company, Sparks, MD, USA) to ensure optimal growth.

For bacterial molecular identification, genomic DNA was extracted using the cetyltrimethylammonium bromide (CTAB) method [53]. Once high-quality and purified DNA was obtained, sequencing was performed by BioSequence, Quito, Ecuador, using Illumina sequencing technology. Molecular identification was not performed for the fungal isolates.

The bacterial sequencing data were processed using Ubuntu 23.04 (Lunar Lobster), a Linux-based operating system. Bioinformatic analysis included file processing, sequence trimming, quality control, and assembly using Trim Galore (version 0.6.10) and Unicycler (version 0.5.1). The assembled sequences were compared with sequences available in the GenBank database using the National Center for Biotechnology Information Basic Local Alignment Search Tool (NCBI BLAST) web version; https://blast.ncbi.nlm.nih.gov/Blast.cgi (accessed on 17 June 2026). In addition, a phylogenetic tree was constructed using Molecular Evolutionary Genetics Analysis (MEGA) software (MEGA 11, version 11.0.13) [54] to evaluate the evolutionary relationships between the bacterial isolates and closely related microorganisms.

### 2.7. Evaluation of Antimicrobial Activity

Antimicrobial activity against the selected bacterial and fungal isolates was evaluated using the agar well diffusion method, following the protocol reported by Olmedo Fonseca (2019) [55], with modifications in the inoculum preparation.

The selected bacterial isolates were cultured in Nutrient Broth, whereas the fungal isolates were cultured in Potato Dextrose Broth (PDB). Cultures were incubated for 24 h under appropriate growth conditions. Subsequently, 100 µL of each bacterial suspension was inoculated onto Petri dishes containing Nutrient Agar, while 100 µL of each fungal suspension was inoculated onto Petri dishes containing Potato Dextrose Agar (PDA). The inoculum was uniformly distributed over the agar surface using sterile swabs [56].

For the agar well diffusion assay, each Petri dish was divided into four quadrants, and one well was prepared in the center of each quadrant using the sterile tip of a Pasteur pipette. Then, 50 µL of CuI nanoparticle suspension was added to the wells at three nominal CuI-equivalent concentrations: high, medium, and low, corresponding to 0.014, 0.0087, and 0.0035 mol/L, respectively. The fourth well was used as the negative control and contained Type I water produced using a Direct-Q^®^ water purification system (Merck KGaA, Darmstadt, Germany). The plates were incubated at 38 °C for 24 h for bacterial assays and at 28 °C for 48 h for fungal assays. All experiments were performed in triplicate. The inhibition zones were measured over a 6-day period to monitor antimicrobial activity over time.

In addition, the antimicrobial activity of banana passion fruit extract without nanoparticles was evaluated under the same experimental conditions. Briefly, 50 µL of extract at three concentrations, C1 = 200 mg/mL, C2 = 100 mg/mL, and C3 = 50 mg/mL, was added to wells 1, 2, and 3, respectively, while Type I water was used as the negative control. Incubation conditions and inhibition-zone measurements were performed as described above.

### 2.8. Cytotoxicity Assay (MTT)

The cytotoxic potential of copper(I) iodide nanoparticles (CuI NPs) was assessed using the colorimetric 3-(4,5-dimethylthiazol-2-yl)-2,5-diphenyltetrazolium bromide assay (MTT; Invitrogen, Eugene, OR, USA). This assay estimates mitochondrial metabolic activity through the reduction in MTT to insoluble formazan crystals and therefore provides an indirect measure of cell viability [57,58,59,60]. Human foreskin fibroblasts (HFF), human breast adenocarcinoma cells (MCF7), and human glioblastoma cells (U251) were used as experimental cell models. Cells were maintained in Dulbecco’s Modified Eagle Medium (DMEM; HiMedia Laboratories, Thane, India) supplemented with 10% fetal bovine serum (FBS; Capricorn Scientific, Ebsdorfergrund, Germany) and 1% penicillin–streptomycin (Corning, Manassas, VA, USA) at 37 °C under a humidified atmosphere containing 5% CO_2_, according to previously reported procedures [58].

For the assay, cells were seeded in 96-well plates (Corning, Kennebunk, ME, USA) at a density of 1 × 10^4^ cells per well and allowed to attach before treatment. The cells were then exposed for 24 and 48 h to nominal CuI-equivalent concentrations of 5.25, 2.63, 1.31, 0.656, 0.331, 0.163, 0.084, 0.042, 0.021, and 0.0105 µmol/L. Four nanoparticle fractions were evaluated: 0.1 mol/L residual, 0.1 mol/L precipitate, 1.0 mol/L residual, and 1.0 mol/L precipitate. These labels refer to the precursor molarity used during synthesis and to the corresponding residual or precipitated fractions. However, the concentrations used in the biological assays are expressed as nominal CuI molar concentrations.

To minimize potential nanoparticle-related interference with the colorimetric assay, blank wells containing culture medium, nanoparticles, and MTT, but no cells, were included. Their absorbance values were subtracted from the corresponding experimental readings [61,62]. After treatment, MTT solution at 0.5 mg/mL in phosphate-buffered saline (PBS; Eurobio Scientific, Les Ulis, France) was added to each well and incubated for 4 h. The resulting formazan crystals were dissolved in dimethyl sulfoxide (DMSO; Fisher Chemical, Geel, Belgium), and absorbance was measured at 570 nm [57,58,59,60].

Cell viability was calculated relative to untreated control cells and expressed as percentage viability. According to International Organization for Standardization (ISO) 10993-5, viability values below 70% were considered indicative of cytotoxicity, while recognizing that this threshold may be influenced by methodological variables [63,64]. Untreated cells were used as the negative control, whereas calcium chloride (CaCl_2_) was used as the positive control to verify assay responsiveness.

**Figure 1:**
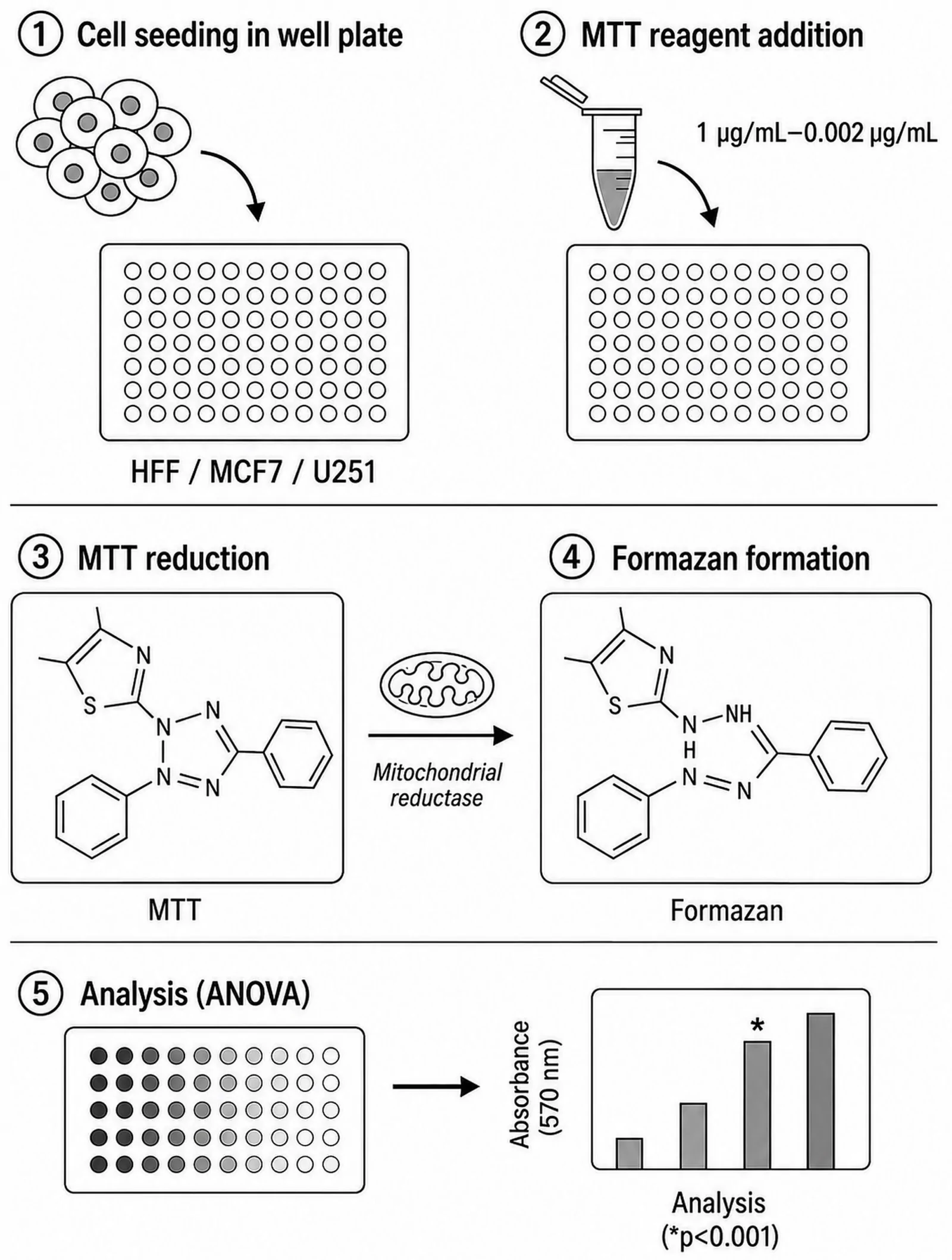
Cytotoxicity (MTT) assay of CuI nanoparticles capped with taxo extract.

Before statistical analysis, residuals were examined to verify model assumptions. When normality assumptions were not met, percentage data were transformed using an appropriate proportion-based transformation [58]. A two-way analysis of variance (ANOVA) was then performed using concentration and exposure time as fixed factors, followed by multiple comparisons against the untreated control. Statistical significance was established at *p* < 0.001. The experimental workflow of the MTT assay is summarized in Figure 1.

### 2.9. Compressive Strength Determination in Reinforced-Concrete Cores (ASTM C1231 and ASTM C39)

The compressive strength of cores extracted from reinforced concrete was evaluated to assess the potential effect of copper(I) iodide nanoparticle (CuI NP) suspensions applied as antifungal and antibacterial treatments. Core extraction, handling, and preparation were performed according to standardized procedures for drilled concrete cores (ASTM International, 2020) [65]. A total of 11 cylindrical cores were tested. For each specimen, the mass and dimensions, including height and diameter, were recorded, and the corresponding cross-sectional area was calculated [65,66].

The cores were subjected to axial compression until failure, following the standard method for compressive strength testing of cylindrical concrete specimens. The maximum applied load was recorded for each specimen (ASTM International, 2023a) [67]. Compressive strength was calculated by dividing the maximum load (P) by the cross-sectional area (A), and the results were expressed in kg/cm^2^ [66,67].

When required, end preparation and capping were performed according to the standard practice for unbonded caps (ASTM International, 2023b) [68]. Correction factors associated with the length-to-diameter ratio (L/D) and the presence of embedded reinforcement were considered equal to 1.00, as reported in the laboratory test report. Nevertheless, both the L/D ratio and the presence of reinforcement are recognized as factors that may influence the measured compressive strength of drilled cores and should therefore be considered when interpreting the results [65,69,70].

For descriptive comparison, the specimens were grouped according to treatment condition: specimens without nanoparticles, distilled-water control, CuI NPs at 1.0 mol/L, and CuI NPs at 0.1 mol/L. This grouping allowed the potential influence of CuI NP treatment on the compressive performance of the concrete cores to be evaluated.

## 3. Results and Discussion

### 3.1. Fruit Extract Analysis (Taxo/Banana Passion Fruit Extract)

The antioxidant activity of the taxo, or banana passion fruit, extract was first evaluated using the DPPH assay, yielding a value of 65.63 ± 4 μmol Trolox g^−1^ fresh weight (FW). This antioxidant capacity supports the presence of redox-active phytochemicals, particularly polyphenolic compounds, which may participate in nanoparticle formation and stabilization [71]. Polyphenols are able to scavenge free radicals and form stable chelates with transition-metal ions, thereby contributing to controlled nucleation, growth regulation, and improved colloidal stability. This behavior is consistent with previous reports indicating that polyphenols can form stable metal complexes through chelation with ions such as cobalt, copper, and magnesium [72].

For Fourier-transform infrared spectroscopy (FTIR) analysis, the aqueous banana passion fruit extract was freeze-dried to obtain a stable dry powder. The lyophilized extract was analyzed by ATR-FTIR in absorbance mode, and the spectra were expressed as absorbance, log(1/T). Spectral preprocessing included smoothing and baseline correction, while band positions were determined by automated peak picking. The FTIR spectrum showed characteristic bands associated with oxygenated functional groups and aromatic or phenolic structures.

The band observed at approximately 2922 cm^−1^ was assigned to aliphatic ν(C–H) stretching vibrations from –CH_2_ and –CH_3_ groups. The signal at approximately 1591 cm^−1^ was attributed to aromatic C=C stretching, suggesting the presence of phenolic or other aromatic structures. The band at approximately 1398 cm^−1^ may be associated with δ(C–H) deformation and/or symmetric ν(COO^−^) stretching. The feature at approximately 1259 cm^−1^ is consistent with C–O stretching vibrations of phenolic or ester groups. In addition, the intense band at approximately 1026 cm^−1^ was assigned to ν(C–O) and ν(C–O–C) vibrations, commonly associated with glycosidic and polysaccharide structures. The band at approximately 775 cm^−1^ corresponds to aromatic C–H out-of-plane bending, whereas the low-wavenumber signals below 1000 cm^−1^ were interpreted as part of the fingerprint region. Weak signals in the 2332–2111 cm^−1^ range were attributed to atmospheric or instrumental contributions, particularly CO_2_, and were therefore not considered diagnostic bands of the extract.

**Figure 2:**
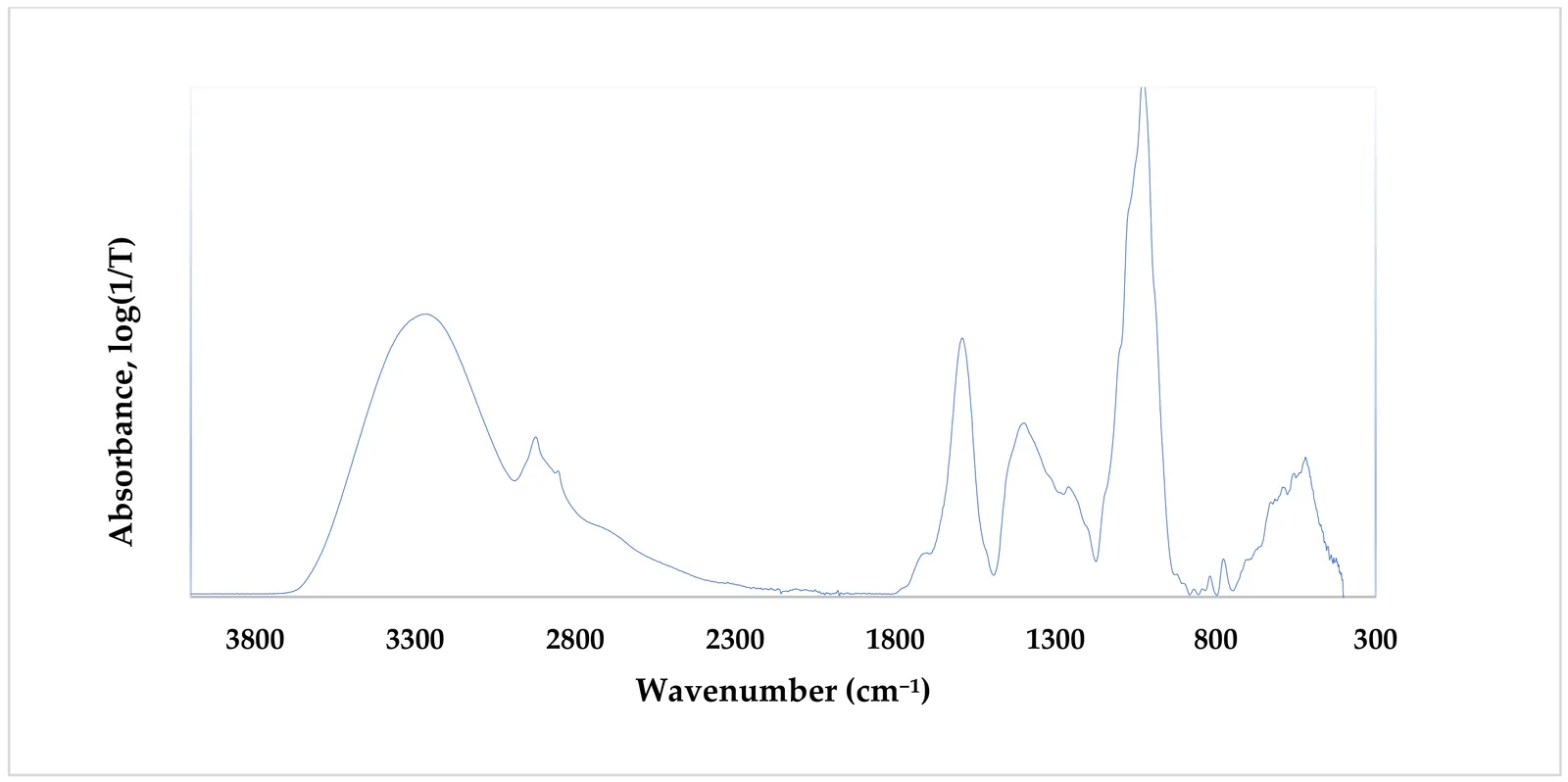
FTIR spectrum of the lyophilized aqueous taxo extract (banana passion fruit) acquired by ATR-FTIR. Absorbance is reported as log(1/T).

Overall, the FTIR results provide evidence of representative functional groups in the banana passion fruit extract, including oxygenated, aromatic/phenolic, carboxylate, and glycosidic or polysaccharide-related moieties. These chemical functionalities may provide coordination and hydrogen-bonding sites, supporting their possible role in metal-ion interaction, surface capping, and stabilization during the green synthesis of CuI nanoparticles [26,73,74,75,76] (Figure 2 and Table 1).

However, it should be noted that FTIR analysis does not allow complete identification or structural elucidation of the chemical composition of the banana passion fruit extract, because the extract is a complex phytochemical mixture. Therefore, the observed FTIR bands were interpreted only as evidence of functional groups potentially involved in nanoparticle formation and stabilization. Detailed identification of individual phytochemical compounds would require complementary analyses, such as NMR and/or LC–MS, which were beyond the scope of the present study.

### 3.2. Structural and Morphological Analysis (UV–Vis Discussion)

**Figure 3:**
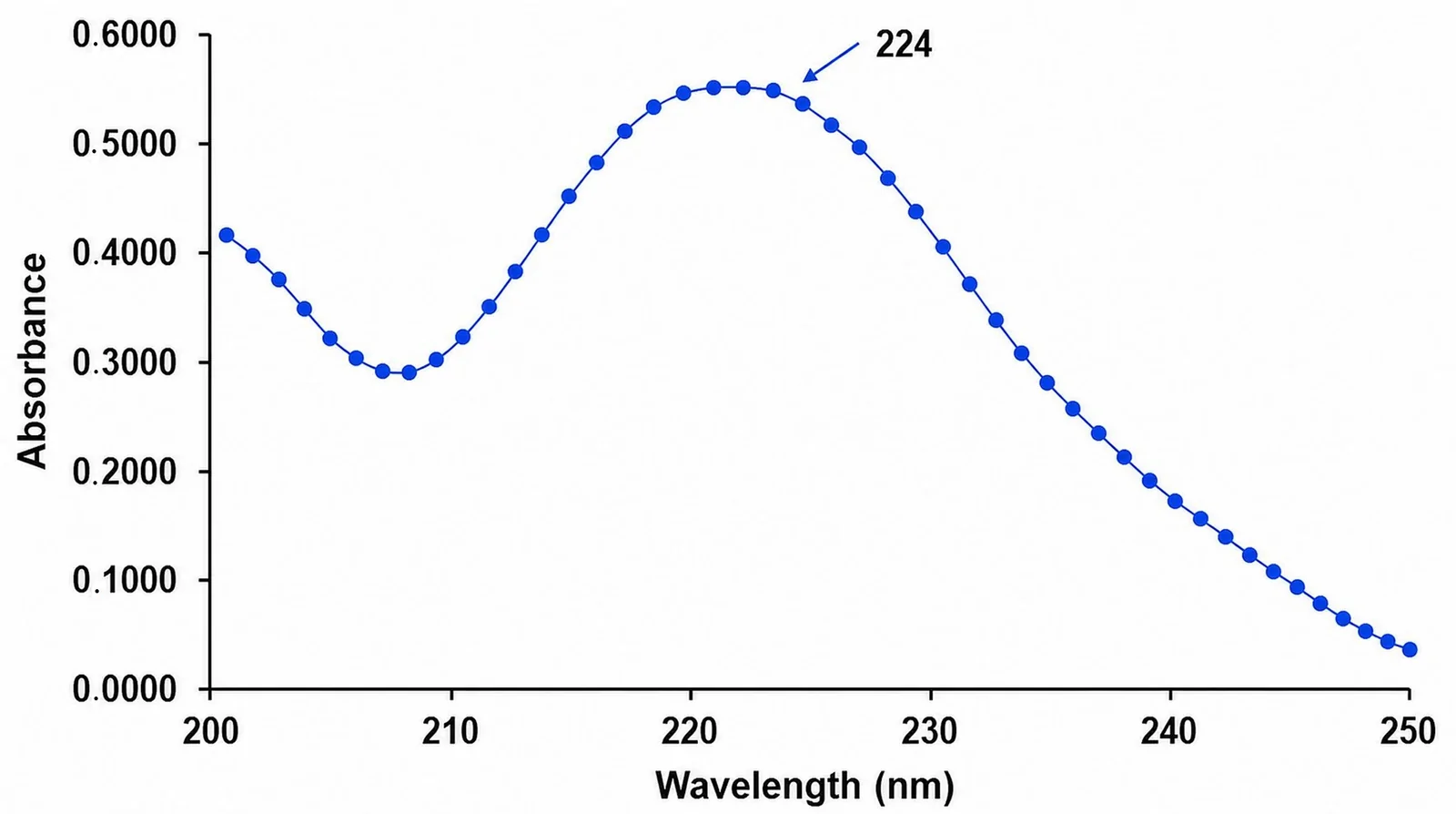
UV–Vis spectrum of the nanoparticles synthesized with taxo extract.

The UV–Vis spectrum of the CuI nanoparticle dispersion synthesized using taxo, or banana passion fruit, extract exhibited an intense absorption band in the UV region, with a maximum at approximately 224 nm (Figure 3). This optical response may appear unusual because the spectrum of the colloidal dispersion can include combined contributions from nanostructured CuI, extract-derived organic compounds adsorbed onto the nanoparticle surface, and light-scattering effects associated with suspended particles. Since the UV–Vis spectrum of the extract alone was not available, the band observed at 224 nm was interpreted conservatively as the overall optical response of the nanoparticle–organic interface rather than as an exclusive signature of CuI nanoparticles. Therefore, phase confirmation of CuI was primarily supported by complementary structural, morphological, and compositional analyses, including XRD, SEM–EDS, and TEM. In this context, UV–Vis spectroscopy was used as a supporting technique to describe the optical behavior of the colloidal dispersion, while avoiding overinterpretation of the absorption band.

The X-ray diffraction (XRD) pattern of the synthesized nanoparticles, recorded using Cu Kα radiation, confirmed the predominant formation of copper(I) iodide (CuI). The main diffraction peaks observed at 2θ ≈ 25.4°, 29.4°, 42.2°, and 49.9° were indexed to the (111), (200), (220), and (311) crystallographic planes, respectively. These reflections are in good agreement with reported reference patterns for CuI and match Joint Committee on Powder Diffraction Standards/International Centre for Diffraction Data (JCPDS/ICDD) card No. 82–2111, confirming the crystallographic identity of the synthesized product. Additional reflections at higher diffraction angles, including those located at approximately 52°, 62°, 67–69°, 77°, and 83°, further support the formation of the γ-CuI phase, which crystallizes under ambient conditions in a cubic zinc-blende structure with space group F-43m.

The sharp and intense diffraction peaks indicate a high degree of crystallinity in the as-prepared nanoparticles. This result is consistent with previous reports on green-synthesized CuI nanoparticles, in which well-defined cubic diffraction patterns and minimal contributions from secondary crystalline phases have been observed. The preservation of the crystalline CuI phase also suggests that banana passion fruit-derived phytochemicals may contribute to the green-synthesis process without disrupting phase formation. Functional groups such as hydroxyl, phenolic, and carboxyl moieties may act as reducing, stabilizing, and capping agents, promoting controlled nucleation and growth while maintaining the crystalline structure of CuI.

**Figure 4:**
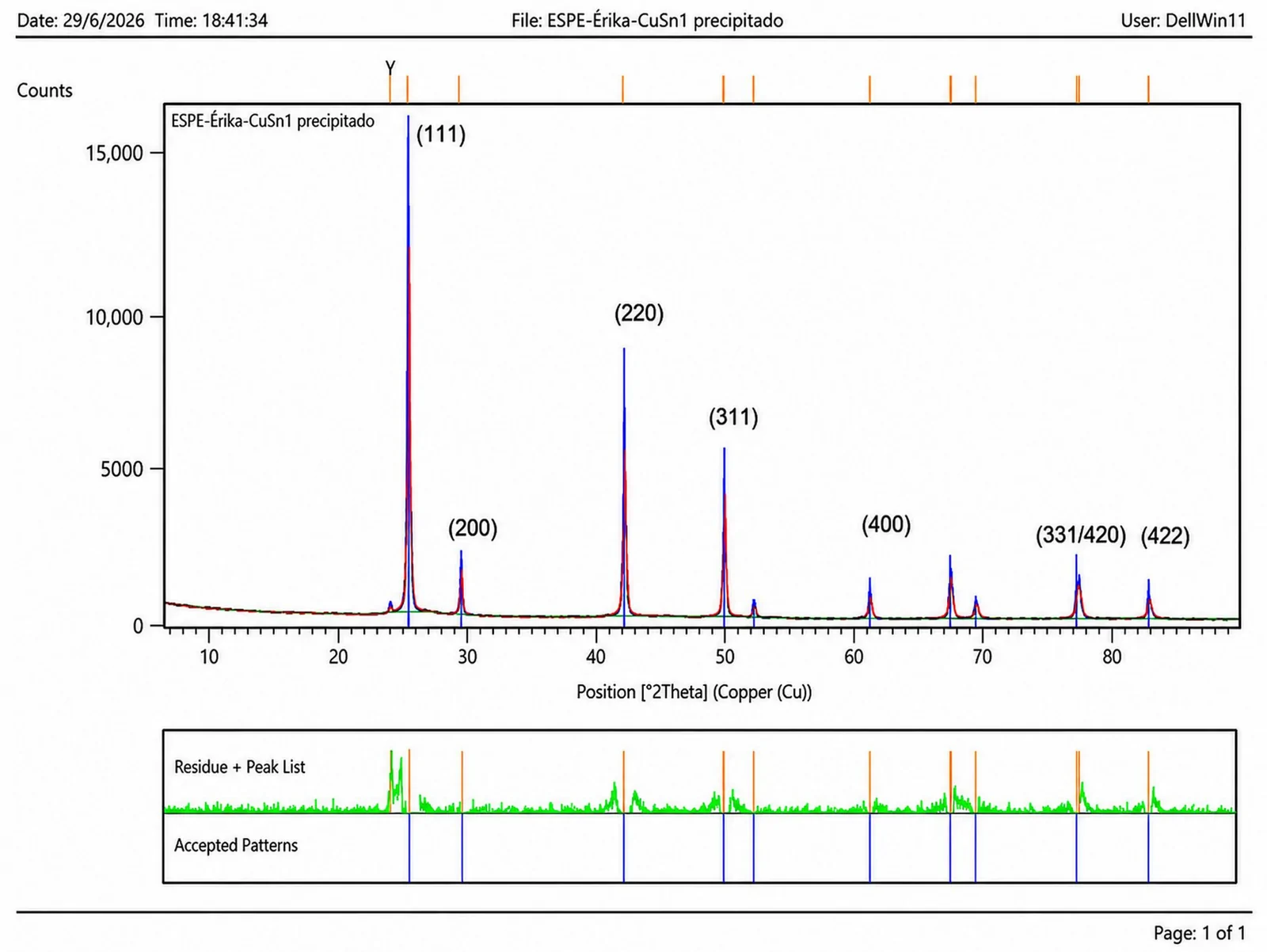
XRD pattern of CuI nanoparticles synthesized with taxo extract, analyzed using HighScore Plus software, version 3.0e (3.0.5) (PANalytical B.V., Almelo, The Netherlands). The green trace in the lower panel represents the residual signal, the blue vertical bars indicate the accepted reference pattern, and the orange markers show the reference peak positions. The diffraction peaks were indexed to cubic copper(I) iodide according to PDF card No. 01-082-2111.

A weak feature near 2θ ≈ 24° was also observed. This signal may be associated with trace amorphous material, residual organic matter from the extract, or a minor non-dominant phase. However, a definitive assignment of this feature would require further analysis, such as systematic comparison with possible impurity patterns and/or Rietveld refinement. Therefore, this minor signal was interpreted cautiously and does not affect the predominant identification of γ-CuI as the main crystalline phase (Figure 4) [37,38,39,40].

TEM analysis confirmed the formation of copper(I) iodide nanoparticles (CuI NPs) with a predominantly spherical morphology and relatively homogeneous dispersion, suggesting that particle nucleation and growth were influenced by the green-synthesis conditions. The presence of residual organic material on the nanoparticle surface suggests the possible formation of a coating derived from banana passion fruit metabolites, which may act as capping and stabilizing agents. This organic layer may contribute to growth control, improved dispersion, and reduced particle agglomeration.

Particle-size analysis was performed using Fiji/ImageJ (ImageJ version 1.54p; National Institutes of Health, Bethesda, MD, USA) and showed nanoparticle diameters ranging from 13 to 45 nm, with most particles concentrated within the intermediate-size range and a smaller fraction extending toward larger diameters. This distribution indicates moderate polydispersity, which is commonly observed in biogenic nanoparticle systems. The difference between the primary particle size measured by TEM and the hydrodynamic diameter determined by DLS, approximately 32 nm, can be attributed to the presence of an organic corona and the solvation layer surrounding the particles in suspension.

**Figure 5:**
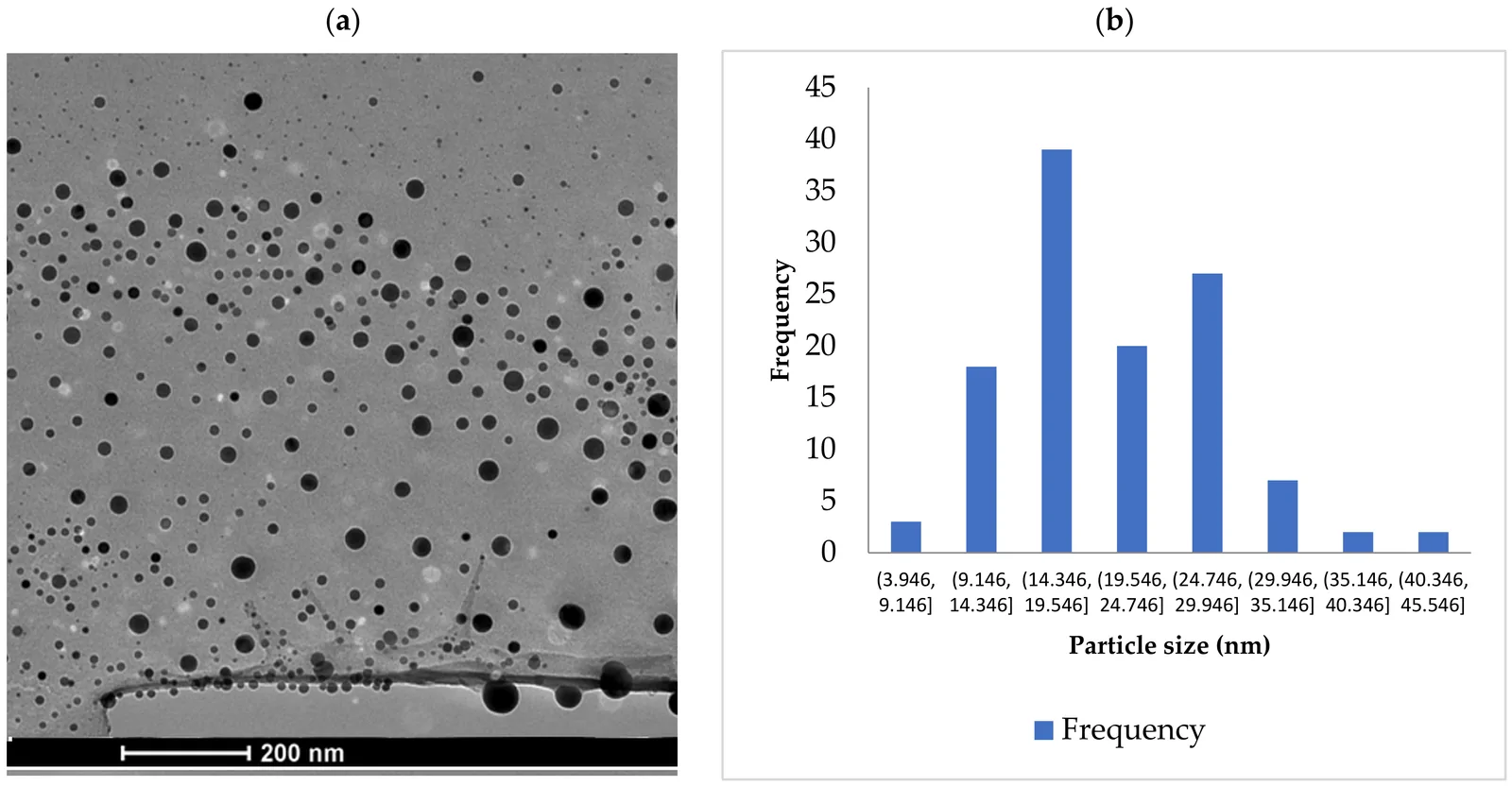
(**a**) TEM images of CuI NPs at different scales in nm. (**b**) Histogram of NP size.

Overall, the observed size, morphology, and dispersion characteristics support the potential antimicrobial performance of CuI NPs against microorganisms isolated from medium-strength concrete. Their nanometric dimensions may provide a high surface area and favor effective interactions with microbial cell structures. This behavior is consistent with mechanisms reported for copper-based nanoparticles, including membrane disruption, ion release, and oxidative stress induction [12,13,14] (Figure 5a,b).

**Figure 6:**
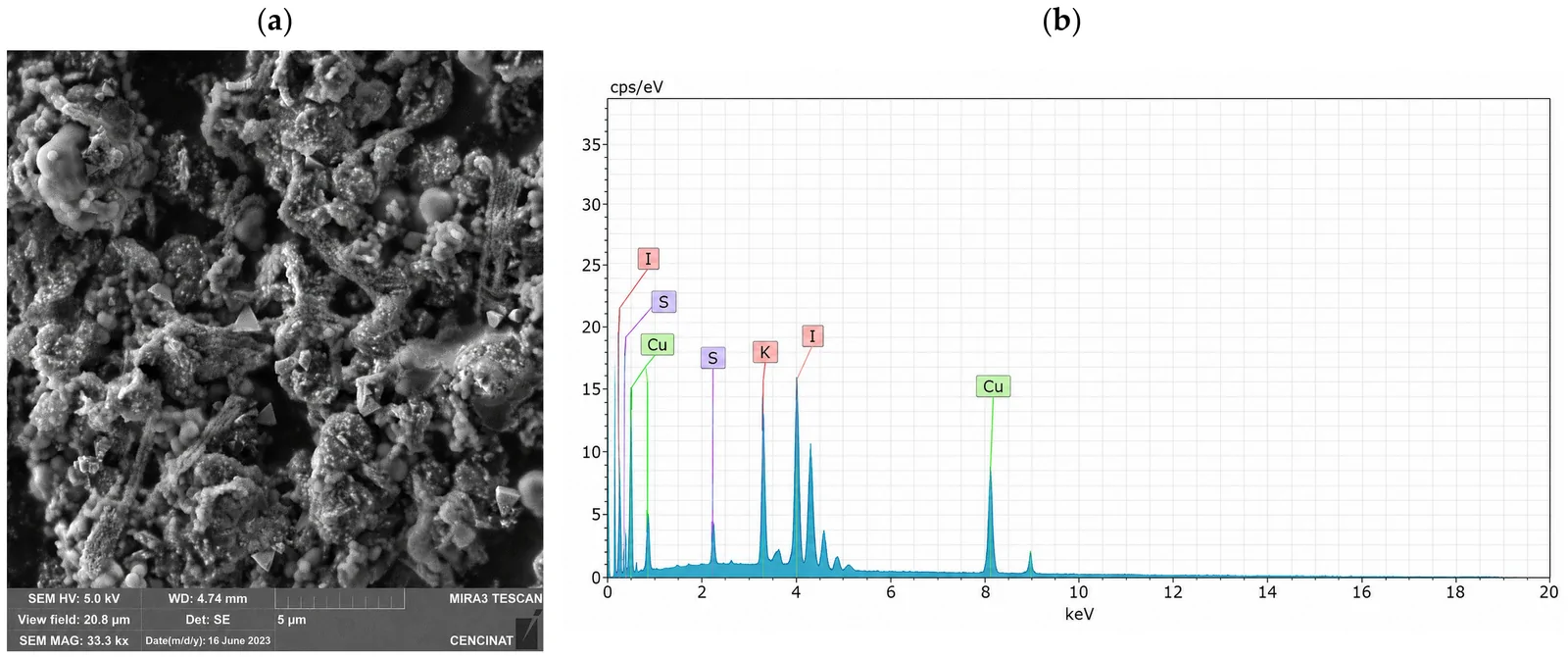
(**a**) SEM micrograph of CuI NPs synthesized via green chemistry using taxo extract (Banana passion fruit), (**b**) EDS spectrum confirming the presence of Cu and I, with minor signals attributed to O, K, and S.

SEM micrographs (Figure 6a) revealed a rough and micro-aggregated morphology in the dry state. This aggregation may be attributed to nanoparticle association during solvent evaporation, capillary forces generated during drying, and the possible bridging effect of banana passion fruit-derived organic compounds adsorbed on the particle surface. Therefore, the micro-aggregated morphology observed by SEM should not be considered contradictory to the TEM results. TEM analysis was performed on a diluted colloidal suspension deposited onto a carbon-coated grid and mainly revealed the primary nanometric CuI particle cores. In addition, the organic coating may be less evident in TEM because extract-derived organic matter has lower electron density than Cu and I. Thus, TEM and SEM provide complementary information: TEM supports the presence of nanometric primary particles, whereas SEM reflects the aggregation behavior of the material after drying.

The EDS spectrum and elemental mapping confirmed the presence and spatial distribution of Cu and I throughout the analyzed region, supporting the formation of copper(I) iodide nanoparticles. The semi-quantitative EDS results are presented in Table 2. Iodine was the most abundant element, with 62.11 normalized wt.% and 30.53 normalized at.%, followed by copper, with 19.36 normalized wt.% and 19.01 normalized at.%. Minor signals corresponding to O, K, and S were also detected. Oxygen may be associated with oxygenated functional groups from banana passion fruit-derived biomolecules adsorbed on the nanoparticle surface, whereas K and S may originate from residual species related to the KI and CuSO_4_ precursors used during synthesis.

Because EDS is a semi-quantitative technique and does not provide direct phase identification, the oxygen signal should not be interpreted as conclusive evidence of copper oxide formation. Therefore, the EDS results were interpreted together with the XRD pattern, which confirmed CuI as the predominant crystalline phase [44,77,78].

No control synthesis without banana passion fruit extract was included in the original experimental design. Therefore, the specific influence of the extract on nucleation and particle growth could not be directly compared with an extract-free synthesis. Nevertheless, the role of the extract as a potential capping and stabilizing medium was inferred from the FTIR evidence of oxygenated and aromatic/phenolic functional groups, together with the structural, morphological, and compositional characterization of the synthesized CuI nanoparticles.

Although FTIR and SEM–EDS analyses support the presence of banana passion fruit-derived organic residues associated with the CuI nanoparticles, these techniques do not allow accurate quantification of the organic-to-inorganic ratio. Therefore, the organic coating was discussed qualitatively as a possible capping and stabilizing contribution rather than as a quantitatively determined component. A precise estimation of the organic/inorganic composition would require complementary analyses, such as TGA, ICP-OES/ICP-MS, XPS, or CHNS elemental analysis.

### 3.3. Molecular Identification

**Figure 7:**
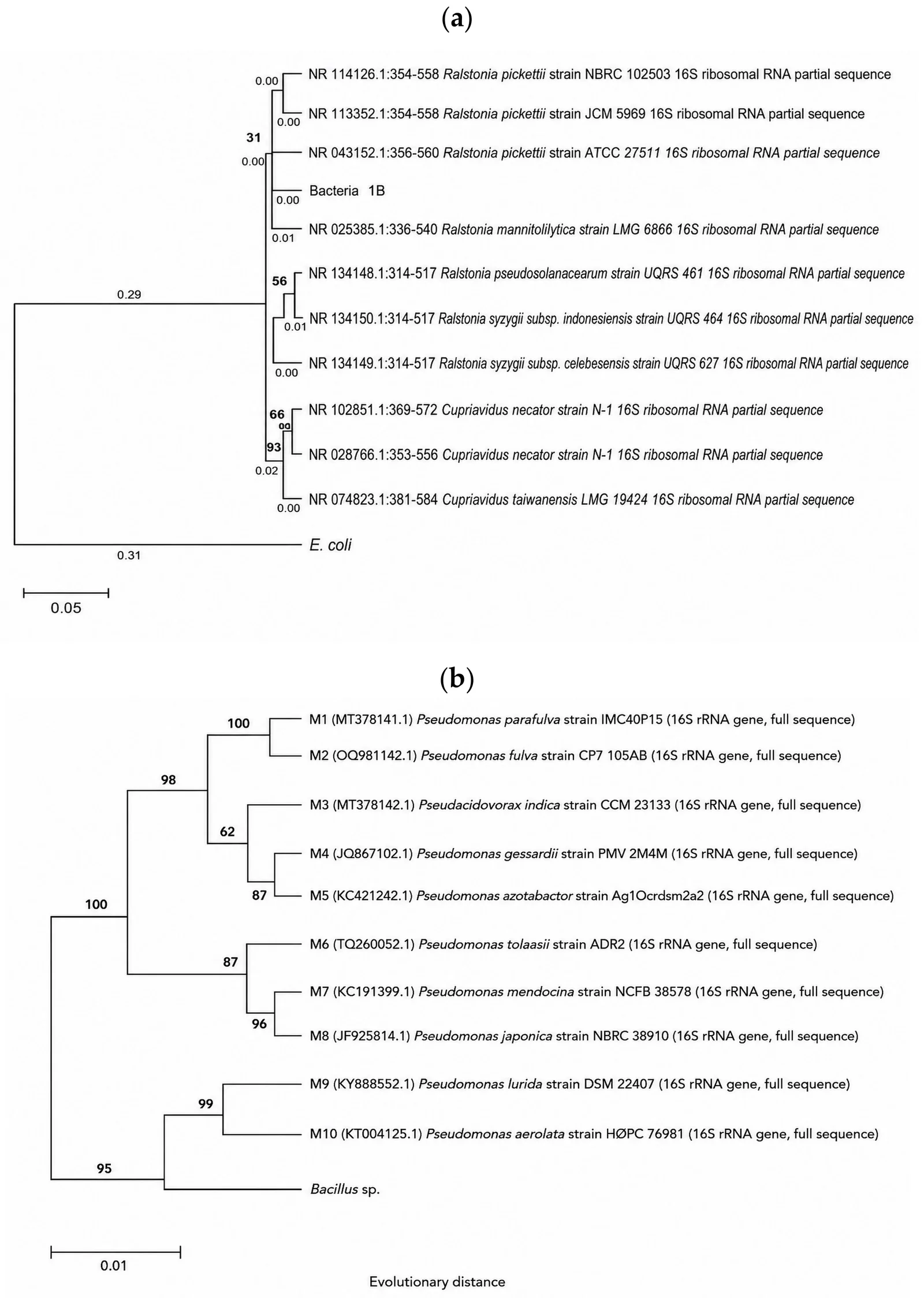
(**a**) Phylogenetic tree for bacterium 1B. The tree reflects the position of the bacterium relative to other similar microorganism, (**b**) Phylogenetic tree for bacterium 2B. The tree reflects the position of the bacterium relative to other similar microorganisms.

The sequences provided by BioSequence, together with the subsequent bioinformatic and phylogenetic analyses, enabled the identification of the bacterial isolates at the genus and species levels as *Ralstonia pickettii* (1B) and *Pseudomonas azotoformans* (2B), respectively. Figure 7a,b present the phylogenetic trees generated in MEGA for isolates 1B and 2B. The numbers shown at each cluster node correspond to bootstrap values obtained from 1000 replicates, and the branches represent microorganisms showing 99% sequence similarity.

Based on phylogenetic analysis, the isolated microorganisms denoted as (1B and 2B) were identified as the Gram-negative bacteria *Ralstonia pickettii* and *Pseudomonas azotoformans*, respectively.

### 3.4. Application of Taxo Extract and CuI Nanoparticles to Isolated Microorganisms

The banana passion fruit extract without nanoparticles did not exhibit antimicrobial activity under the tested conditions. In contrast, the CuI nanoparticles synthesized using the extract showed clear antimicrobial inhibition against both bacterial and fungal isolates. A previous study on seed oils extracted from yellow passion fruit (*Passiflora edulis* var. *flavicarpa*), a species belonging to the same genus, reported high antioxidant performance and antibacterial activity against *Escherichia coli*, *Salmonella enteritidis*, *Staphylococcus aureus*, and *Bacillus cereus* [79]. However, in the present study, the banana passion fruit extract alone did not show antimicrobial activity, whereas the extract-mediated CuI nanoparticles exhibited evident inhibitory effects. This finding suggests that the antimicrobial performance observed in this work is mainly associated with the CuI nanoparticle system rather than with the free extract itself.

The antimicrobial activity of CuI nanoparticles at three nominal concentrations, 0.014, 0.0087, and 0.0035 mol/L, was evaluated against the two Gram-negative bacterial strains by measuring inhibition-zone diameters using an agar diffusion-based method [56]. Inhibition zones were observed against *R. pickettii* at all tested nanoparticle concentrations (Table 3).

**Figure 8:**
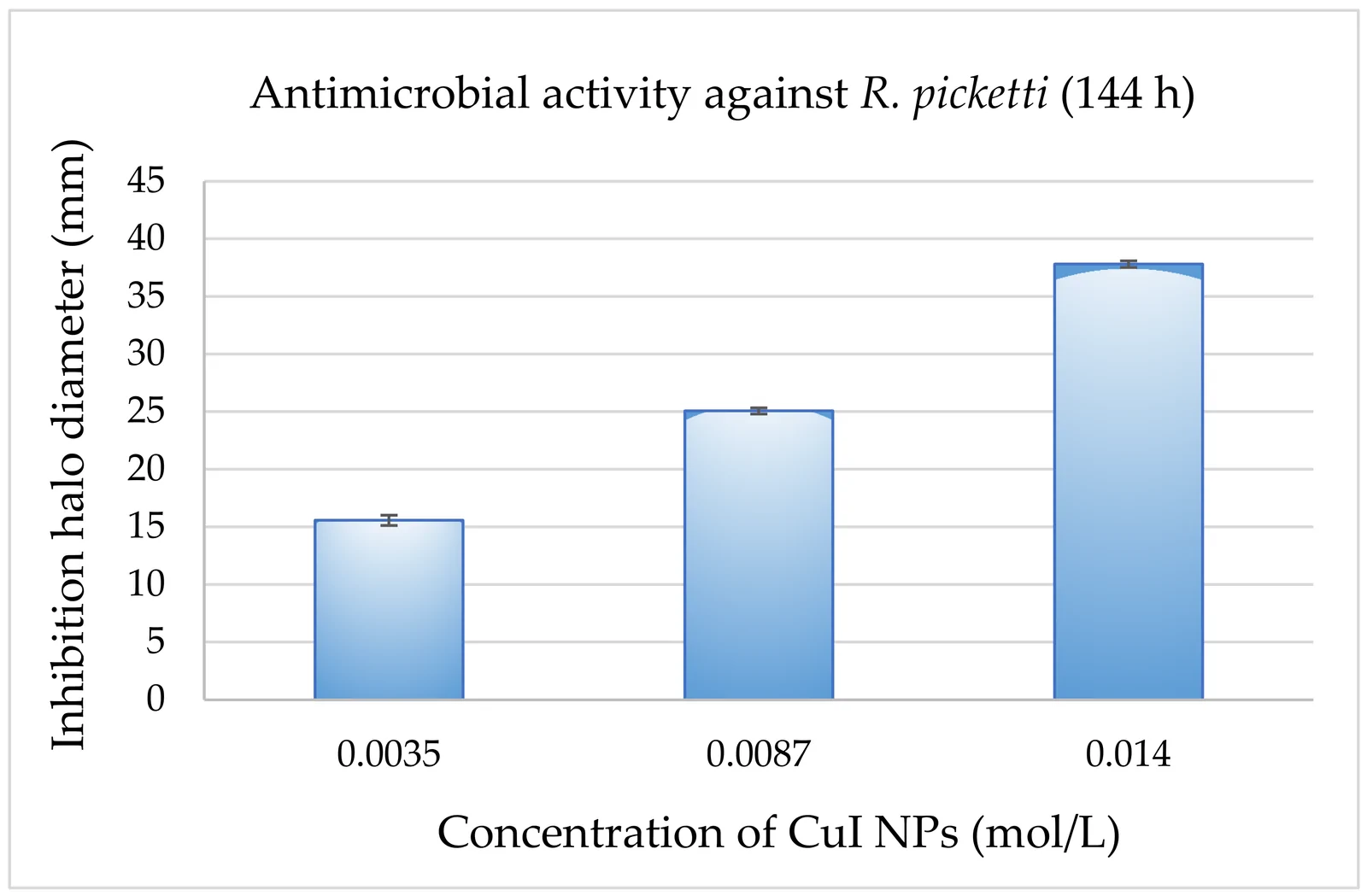
Quantitative analysis of inhibition-zone diameters produced by CuI NPs against *Ralstonia pickettii* after 144 h of exposure. Values are expressed as mean ± standard deviation.

In contrast, *P. azotoformans* showed bacterial growth only around the blank well containing water as the negative control, while no evident growth was observed in the areas treated with CuI NPs. This behavior suggests a marked susceptibility of *P. azotoformans* to the CuI nanoparticle treatment under the tested conditions. In the case of *R. pickettii*, inhibition zones were measurable at all tested concentrations, and Figure 8 summarizes the quantitative analysis of inhibition-zone diameters after 144 h of exposure. These results support the antimicrobial potential of CuI NPs against Gram-negative bacteria isolated from concrete surfaces. They are also consistent with previous findings reporting that copper-based nanoparticles (CBNPs) can exert broad-spectrum antimicrobial activity by damaging key microbial components, including proteins, lipids, and nucleic acids [13].

**Figure 9:**
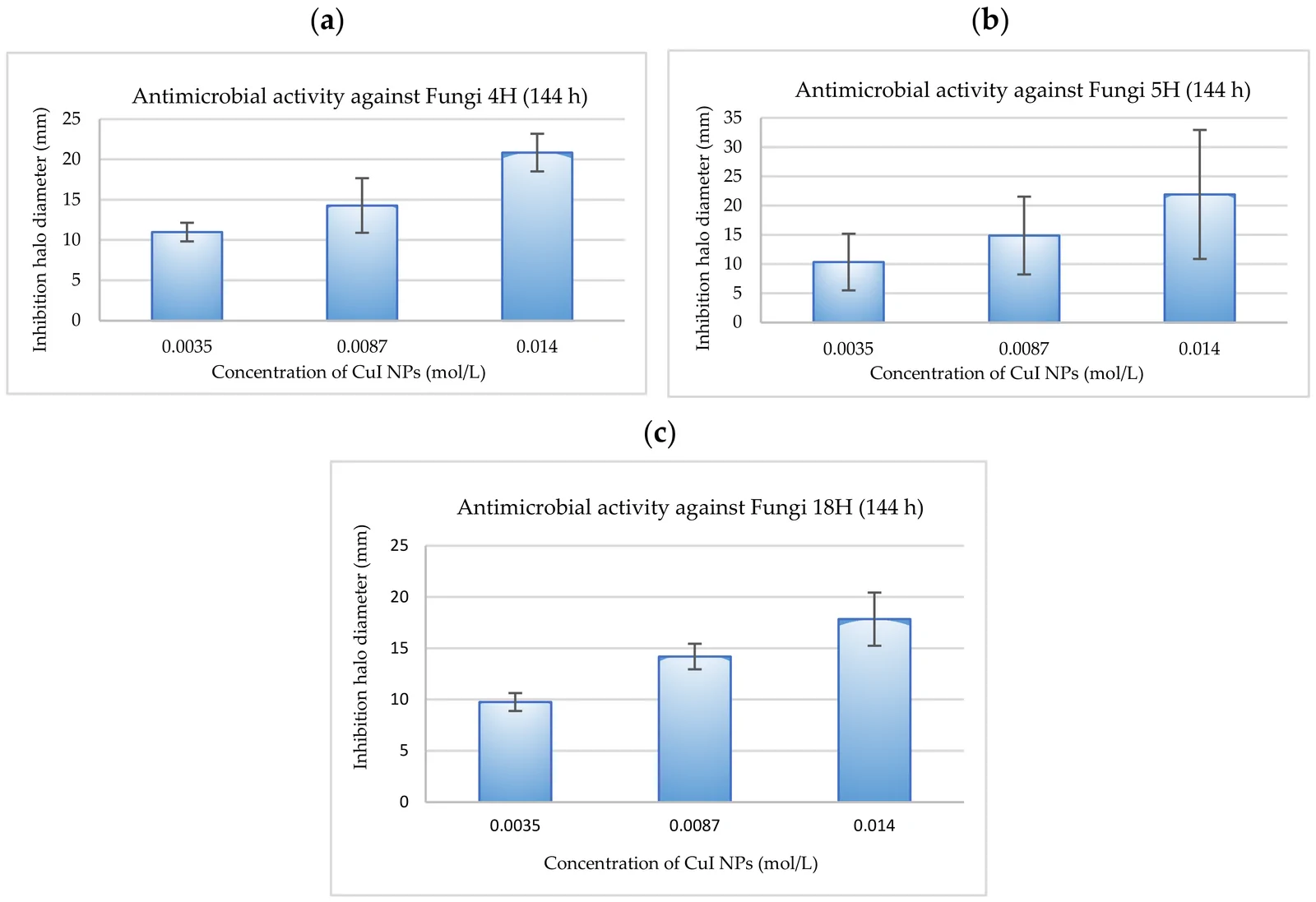
Quantitative analysis of inhibition-zone diameters produced by CuI NPs against fungal isolates after 144 h of exposure: (**a**) fungal isolate 4H; (**b**) fungal isolate 5H; (**c**) fungal isolate 18H. Values are expressed as mean ± standard deviation.

Three selected fungal isolates were coded as 4H, 5H, and 18H. Figure 9 shows the quantitative analysis of inhibition-zone diameters produced by CuI NPs against the fungal isolates 4H, 5H, and 18H after 144 h of exposure (Table 4).

The progressive reduction in inhibition-zone diameters over time may be associated with physicochemical changes in the nanoparticle suspensions, including oxidation, aggregation, and surface-state modifications, which may reduce antimicrobial effectiveness [38,80]. Nevertheless, the agar well diffusion assay confirmed the antimicrobial activity of the banana passion fruit-mediated CuI NPs, with inhibition-zone diameters varying according to nanoparticle concentration. Overall, a concentration-dependent response was observed, with the highest concentration tested, 0.014 mol/L, producing the largest inhibition zones.

The inhibition zones observed for most of the predominant strains isolated from concrete indicate that the nanoparticles were able to diffuse through the agar medium and effectively inhibit microbial growth. This behavior is consistent with reported mechanisms for copper-based and CuI nanoparticles, including ion release, reactive oxygen species (ROS) generation, membrane disruption, and damage to essential biomolecules such as lipids, proteins, and nucleic acids [11,12,13,14]. In particular, Cu^+^/Cu^2+^ redox cycling may enhance oxidative stress, while direct interactions between nanoparticles and microbial membranes can increase membrane permeability and promote leakage of intracellular components. These combined mechanisms may explain the broad-spectrum antimicrobial performance reported for CuI-based systems against both Gram-positive and Gram-negative bacteria, including resistant strains [14,17,18].

Previous studies have also shown that green-synthesized CuI NPs obtained using natural extracts, such as anthocyanin-rich red cabbage extract, exhibit improved colloidal stability due to organic capping and significant antimicrobial activity against *Escherichia coli* and *Staphylococcus aureus*. These systems commonly show a dose-dependent response, with higher nanoparticle concentrations producing stronger antibacterial effects [81]. A similar trend was observed in the present study, supporting the role of nanoparticle concentration as a key factor controlling the antimicrobial performance of banana passion fruit-mediated CuI NPs.

Despite these advances, limited information is available on the incorporation of green-synthesized CuI NPs into cementitious matrices. Most previous studies have focused on immobilization onto solid substrates, such as textiles, where in situ CuI NP deposition has achieved microbial reductions greater than 99.9% against bacteria, fungi, and viruses within short contact times of 2–5 min [21,22,82]. Compared with these systems, the results obtained in this work suggest that CuI NPs can retain antimicrobial functionality when applied to more complex mineral-based materials.

Therefore, extending this immobilization strategy to cement and concrete matrices represents a promising route for developing multifunctional materials with enhanced resistance to microbial colonization and biodeterioration. The antimicrobial activity observed in this study supports the feasibility of using banana passion fruit-mediated CuI NPs as additives or functional coatings for cementitious materials, while the green-synthesis approach contributes to the development of more sustainable antimicrobial systems [19,20,21,22,23,82].

### 3.5. Cytotoxicity Assessment by MTT Assay

CuI NPs induced a concentration- and time-dependent reduction in metabolic viability in the three evaluated cell lines—HFF, MCF7, and U251—as determined by the MTT assay. Because this assay provides an indirect measure of mitochondrial metabolic activity, the results should be interpreted as a preliminary cytotoxicity screening rather than as evidence of a specific mechanism of cell death. Microscopic examination showed a progressive decrease in cell density with increasing nanoparticle concentration and exposure time, together with visible morphological alterations. Overall, the formulations obtained under the 1.0 mol/L synthesis condition produced the strongest reduction in metabolic viability, particularly the precipitated fraction. In contrast, the 0.1 mol/L formulations, especially the residual fraction, showed comparatively higher cell viability and more preserved cellular morphology.

In HFF cells, the greatest reduction in viability was observed for the 1.0 mol/L precipitated fraction, followed by the 1.0 mol/L residual fraction. Conversely, the 0.1 mol/L formulations maintained viability values above 70% at concentrations ≤ 0.656 µmol/L, according to the ISO 10993-5 reference criterion [63,64]. In MCF7 cells, metabolic viability decreased markedly at concentrations ≥ 1.31 µmol/L, with an almost complete loss of viability at 5.25 µmol/L after 48 h of exposure. U251 cells showed an intermediate response, with significant reductions in viability from 1.31 µmol/L onward, particularly for the 1.0 mol/L-derived samples. Among the tested formulations, the 0.1 mol/L residual fraction exhibited the highest relative viability across the three cell lines.

However, these findings should not be interpreted as evidence of selective anticancer activity or as confirmation of a specific cytotoxic mechanism. Additional assays, including reactive oxygen species (ROS) generation, lactate dehydrogenase (LDH) release, Annexin V/propidium iodide (PI) staining, and complementary colloidal characterization under cell-culture conditions, would be required to evaluate oxidative stress, membrane damage, apoptosis/necrosis, nanoparticle stability, and potential cell-type-specific responses. In addition, although DLS analysis in aqueous suspension indicated a mean hydrodynamic diameter of approximately 32 nm, zeta-potential measurements and DLS analysis in serum-containing culture medium were not included in the original experimental design. Therefore, no conclusions can be drawn regarding nanoparticle surface charge, protein-corona formation, intracellular uptake, or specific mechanisms of cell death under cell-culture conditions.

**Figure 10:**
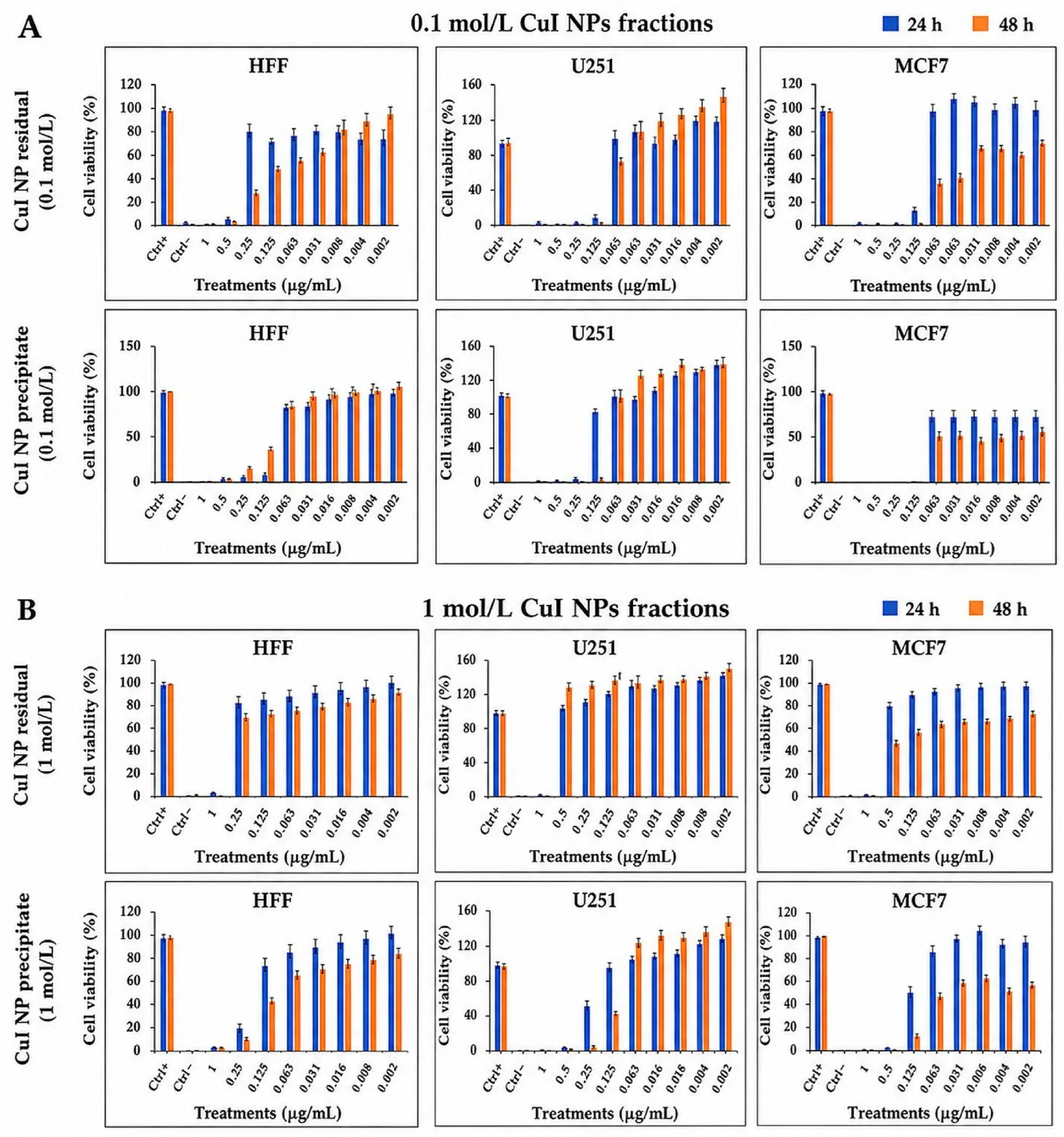
Dose–response cytotoxicity profile of CuI nanoparticle fractions in HFF, U251, and MCF7 cells after 24 and 48 h of exposure, as determined by the MTT assay. (**A**) CuI NP fractions derived from the 0.1 mol/L synthesis condition: residual fraction (upper row) and precipitated fraction (lower row). (**B**) CuI NP fractions derived from the 1.0 mol/L synthesis condition: residual fraction (upper row) and precipitated fraction (lower row). Cell viability is expressed as percentage relative to untreated controls. Data are presented as mean ± standard deviation. Statistical analysis was performed using two-way ANOVA, considering nanoparticle concentration and exposure time as fixed factors, followed by multiple comparisons against the untreated control. Statistical significance was established at *p* < 0.001.

Accordingly, the MTT assay should be considered a preliminary biocompatibility screening intended to support further optimization of CuI NP formulations for antimicrobial applications in cementitious materials, rather than a definitive toxicological or mechanistic assessment (Figure 10).

The MTT assay results indicate that the reduction in metabolic viability induced by copper(I) iodide nanoparticles (CuI NPs) depended on concentration, exposure time, and formulation type, including the synthesis condition, 0.1 mol/L versus 1.0 mol/L, and the analyzed fraction, residual versus precipitated. In particular, the 1.0 mol/L-derived samples, especially the precipitated fraction, produced the greatest reduction in metabolic viability, whereas the 0.1 mol/L residual fraction showed the highest relative viability across the evaluated concentration range. These differences may be associated with variations in nanoparticle concentration, surface chemistry, residual banana passion fruit-derived organic coating, aggregation behavior, and possible release of copper species. However, these factors were not directly quantified in the present study.

The stronger reduction in metabolic viability observed after 48 h suggests a time-dependent response. Nevertheless, this result should not be interpreted as direct evidence of intracellular oxidative stress, membrane disruption, mitochondrial dysfunction, or a specific cell-death pathway. Although these mechanisms have been reported for metallic nanomaterials and copper-based systems [33,34,35,36], their confirmation for the present CuI NP formulations would require complementary assays, including ROS generation, LDH release, Annexin V/PI staining, and copper-release quantification. Similarly, although MCF7 and U251 cells showed lower metabolic viability than HFF cells under some experimental conditions, these differences should not be interpreted as evidence of selective anticancer activity. Since the MTT assay primarily reflects metabolic activity, additional mechanistic and cell-specific assays would be necessary before proposing any selective biological effect [57,58,59,60].

Nanoparticle accumulation or intracellular uptake was not directly evaluated in the present study. Therefore, the observed reduction in metabolic viability should not be interpreted as evidence of CuI NP internalization or accumulation within cells. Confirmation of nanoparticle–cell association or intracellular accumulation would require complementary techniques, such as fluorescence microscopy using labeled nanoparticles, flow cytometry based on side-scatter analysis, or electron microscopy. Accordingly, the MTT assay was considered only as a preliminary cytotoxicity screening, and future studies should include these approaches to better understand nanoparticle uptake, accumulation, and cell-interaction mechanisms.

Overall, the 0.1 mol/L residual CuI formulation showed the most favorable preliminary cytotoxicity profile, preserving comparatively higher metabolic viability under the tested conditions. This result supports its consideration for further optimization as an antimicrobial formulation for cementitious materials. However, additional colloidal characterization under cell-culture conditions, including zeta-potential analysis and time-dependent DLS in serum-containing medium, together with complementary biological assays, will be required to better define nanoparticle stability, cell interactions, accumulation behavior, and safety-related performance.

### 3.6. Mechanical Compatibility: Compressive Strength of Reinforced-Concrete Cores

Reinforced-concrete cores were also evaluated by compressive-strength testing to assess whether the application of CuI NP suspensions affected the mechanical performance of the material. The results showed substantial variability among specimens, with both high and low compressive-strength values observed in the untreated group without nanoparticles and in the groups treated with CuI NP suspensions at 1.0 mol/L and 0.1 mol/L.

Under these conditions, the within-group dispersion and the limited number of replicates per treatment did not allow a robust identification of a consistent monotonic trend, either positive or negative, attributable to CuI NP application. Therefore, within the scope of the present mechanical evaluation, the data suggest that CuI NPs did not produce a consistent effect on the compressive strength of concrete.

Nevertheless, additional replicates, a more balanced experimental design, and statistical analysis with sufficient sample size are required to confirm or rule out subtle differences with statistical significance. These results should therefore be interpreted as a preliminary mechanical compatibility assessment rather than as definitive evidence of the long-term structural effect of CuI NP treatment on concrete cores (Table 5).

## 4. Conclusions

In this work, copper(I) iodide nanoparticles (CuI NPs) were successfully synthesized at room temperature through a green route using banana passion fruit extract. The extract exhibited relevant antioxidant capacity, as determined by the DPPH assay, with a value of 65.63 ± 4 μmol Trolox g^−1^ fresh weight (FW), supporting the presence of redox-active metabolites that may contribute to nanoparticle formation and stabilization. UV–Vis characterization revealed an intense absorption band in the UV region, with λmax ≈ 224 nm. Since the UV–Vis spectrum of the extract alone was not available, this band was interpreted conservatively as the overall optical response of the CuI nanoparticle–organic interface rather than as an exclusive signature of CuI formation.

XRD analysis confirmed that the synthesized product consisted predominantly of highly crystalline γ-CuI with a cubic zinc-blende structure, in agreement with JCPDS/ICDD PDF card No. 01-082-2111, and without prominent reflections attributable to secondary crystalline impurities. FTIR analysis of the dried banana passion fruit extract revealed oxygenated functional groups, including C–O/C–O–C vibrations, contributions consistent with –COO^−^ groups, and aromatic/phenolic signatures. These functional groups support the possible role of extract-derived biomolecules as capping and stabilizing agents during CuI nanoparticle formation. TEM analysis showed mostly spherical nanoparticles with relatively homogeneous dispersion and particle sizes in the 13–45 nm range, indicating moderate polydispersity. The difference between the primary particle size observed by TEM and the hydrodynamic diameter measured by DLS, approximately 32 nm, was attributed to the presence of an organic corona and solvation layer in suspension. In the dry state, SEM revealed micro-aggregation, while EDS confirmed Cu and I as the main elements. Minor O, K, and S signals were attributed to residual organic coating and/or trace precursor-related species. Therefore, oxygen detection by EDS should not be interpreted as direct evidence of copper oxide formation and must be considered together with the XRD results.

From a functional perspective, CuI NPs showed antimicrobial activity against bacteria isolated from medium-strength concrete, including *Ralstonia pickettii* and *Pseudomonas azotoformans*, as well as against unidentified fungal isolates. These findings support their potential application as antimicrobial additives or functional coatings for cementitious materials. However, due to the high variability of the compressive-strength data and the limited number of specimens, the mechanical evaluation should be considered preliminary. Therefore, no definitive conclusion can be drawn regarding the effect of CuI NPs on the mechanical properties of concrete. Preliminary MTT screening showed concentration- and time-dependent reductions in metabolic viability, with stronger effects observed for the 1.0 mol/L formulations, particularly the precipitated fraction. In contrast, the 0.1 mol/L formulations, especially the 0.1 mol/L residual CuI fraction, showed a more favorable preliminary cytotoxicity profile. These findings highlight the need to optimize synthesis conditions to achieve an appropriate balance between antimicrobial efficacy and biological safety.

Future work should strengthen both the physicochemical and biological evaluation of CuI NPs. This should include band-gap estimation through Tauc analysis, colloidal stability studies using ζ-potential measurements and time-dependent DLS under different pH and ionic-strength conditions, copper-release quantification in relevant media by ICP-OES or ICP-MS, and complementary surface characterization by FTIR and XPS. Biological evaluation should be extended beyond the MTT assay to include mechanistic studies, such as ROS generation, LDH release, and Annexin V/PI staining. Although Raman spectroscopy may provide complementary vibrational information, the most diagnostic CuI modes are located in the low-wavenumber region; therefore, when measurements below 200 cm^−1^ are not accessible, structural confirmation should rely primarily on XRD, supported by FTIR, TEM, DLS, and SEM–EDS. In addition, the performance of these nanoparticles should be validated in realistic cementitious matrices by assessing microbial colonization, biofilm formation, mechanical behavior, leaching, and long-term durability under wet–dry cycling, carbonation, and chloride exposure conditions. Future studies should also include an extract-free synthesis control to directly evaluate the influence of banana passion fruit-derived biomolecules on CuI nanoparticle nucleation, growth, surface stabilization, and colloidal stability.

## Figures and Tables

**Table 1:** Representative FTIR bands of the dried (Banana Passion Fruit Extract) extract (ATR; absorbance spectrum, log(1/T); SpectraGryph v1.2.16.1 pre-processing: smoothing and baseline correction).

Band Position (cm^−1^)	FWHM (cm^−1^) *	Tentative Assignment	Interpretation/Relevance
2922.1	271.76	ν_as(C–H) (–CH_2_/–CH_3_)	Aliphatic components; contributes to an organic surface layer
1591.4	80.80	ν(C=C)_ar	Aromatic signal associated with polyphenols/flavonoids
1398.0	64.42	δ(C–H) and/or ν_s(COO^−^)	C–H deformation and/or carboxylate contribution (organic acids)
1259.3	—	ν(C–O) (phenols/esters)	C–O vibrations characteristic of phenolic compounds
1026.6	106.71	ν(C–O), ν(C–O–C)	Intense band from glycosidic/polysaccharide structures (extract “fingerprint”)
775.3	22.09	γ(C–H)_oop	Substituted aromatics (out-of-plane vibration)
518.4	216.73	Fingerprint region (skeletal vibrations)	Complementary/supporting band

* FWHM: full width at half maximum. FWHM is reported when determined by the SpectraGryph peak-picking algorithm; “—” indicates that FWHM was not estimated under the applied detection settings. Note: Weak signals in the 2323–2111 cm^−1^ range were detected and attributed to environmental/instrumental contributions (e.g., CO_2_) and were not considered diagnostic of the extract.

**Table 2:** Semi-quantitative elemental composition obtained by EDS analysis of CuI NPs synthesized using taxo extract.

Element	Series	wt.%	Norm. wt.%	Norm. at.%	Error in wt.%
O	K-series	7.86	8.89	34.65	0.95
S	K-series	1.14	1.29	2.50	0.07
K	K-series	7.38	8.35	13.32	0.25
Cu	K-series	17.12	19.36	19.01	0.46
I	L-series	54.92	62.11	30.53	1.57
Total	—	88.43	100.00	100.00	

**Table 3:** Average values of the diameter of inhibition halos. Mean inhibition-zone diameters produced by CuI NPs against *Ralstonia pickettii* after 144 h of exposure.

Bacteria	Time (Hours)	Concentration of CuI NPs (mol/L)	Average Diameter (mm)	SD
1B		0.0035	15.57	0.45
*R. pickettii*	144	0.0087	25.07	0.27
		0.014	37.81	0.29

SD: Standard deviation.

**Table 4:** Average values of the diameter of inhibition halos. Mean inhibition-zone diameters produced by CuI NPs against fungal isolates 4H, 5H, and 18H after 144 h of exposure.

Fungi	Time (Hours)	Concentration NPs CuI (mol/L)	Average Diameter (mm)	SD
		0.0035	10.99	1.16
4H	144	0.0087	14.29	3.38
		0.014	20.85	2.34
		0.0035	10.35	4.84
5H	144	0.0087	14.88	6.65
		0.014	21.92	11.04
		0.0035	9.76	0.87
18H	144	0.0087	14.20	1.25
		0.014	17.85	2.59

SD: Standard deviation.

**Table 5:** Compressive strength results for reinforced-concrete cores treated with CuI–NP suspensions.

Condition	*n*	Mean Strength ± Standard Deviation (SD) (kgf/cm^2^)	Range (kgf/cm^2^)
Without CuI NPs	3	449.6 ± 245.9	172.7–642.5
Distilled-water control	2	637.0 ± 65.2	590.9–683.1
CuI NPs 1.0 mol/L	3	429.8 ± 101.4	346.1–542.6
CuI NPs 0.1 mol/L	3	341.5 ± 157.2	239.7–522.6

## Data Availability

The original contributions presented in the study are included in the article. Further inquiries can be directed to the corresponding author.

## References

[1] Romani M., Warscheid T., Nicole L., Marcon L., Di Martino P., Suzuki M.T., Lebaron P., Lami R. (2022). Current and future chemical treatments to fight biodeterioration of outdoor building materials and associated biofilms: Moving away from ecotoxic and towards efficient, sustainable solutions. Sci. Total Environ..

[2] Gulotta D., Villa F., Cappitelli F., Toniolo L. (2018). Biofilm colonization of metamorphic lithotypes of a renaissance cathedral exposed to urban atmosphere. Sci. Total Environ..

[3] Li Q., Zhang B., Yang X., Ge Q. (2018). Deterioration-associated microbiome of stone monuments: Structure, variation, and assembly. Appl. Environ. Microbiol..

[4] Pena-Poza J., Ascaso C., Sanz M., Pérez-Ortega S., Oujja M., Wierzchos J., Souza-Egipsy V., Cañamares M.V., Urizal M., Castillejo M. (2018). Effect of biological colonization on ceramic roofing tiles by lichens and a combined laser and biocide procedure for its removal. Int. Biodeterior. Biodegrad..

[5] Joseph E. (2021). Microorganisms in the Deterioration and Preservation of Cultural Heritage.

[6] He D., Wu F., Ma W., Gu J.-D., Xu R., Hu J., Yue Y., Ma Q., Wang W., Li S.-W. (2022). Assessment of cleaning techniques and its effectiveness for controlling biodeterioration fungi on wall paintings of Maijishan Grottoes. Int. Biodeterior. Biodegrad..

[7] Fidanza M.R., Caneva G. (2019). Natural biocides for the conservation of stone cultural heritage: A Review. J. Cult. Herit..

[8] Arreche R., Vázquez P. (2020). Green biocides to control biodeterioration in materials science and the example of preserving World Heritage Monuments. Curr. Opin. Green Sustain. Chem..

[9] Fouda A., Abdel-Maksoud G., Abdel-Rahman M.A., Eid A.M., Barghoth M.G., El-Sadany M.A.-H. (2019). Monitoring the effect of biosynthesized nanoparticles against biodeterioration of cellulose-based materials by *Aspergillus niger*. Cellulose.

[10] Barberia-Roque L., Obidi O.F., Gámez-Espinosa E., Viera M., Bellotti N. (2019). Hygienic coatings with bioactive nano-additives from *Senna occidentalis*-mediated green synthesis. NanoImpact.

[11] Ramos-Zúñiga J., Bruna N., Pérez-Donoso J.M. (2023). Toxicity mechanisms of copper nanoparticles and copper surfaces on bacterial cells and viruses. Int. J. Mol. Sci..

[12] Ma X., Zhou S., Xu X., Du Q. (2022). Copper-containing nanoparticles: Mechanism of antimicrobial effect and application in dentistry-a narrative review. Front. Surg..

[13] Li X., Cong Y., Ovais M., Cardoso M.B., Hameed S., Chen R., Chen M., Wang L. (2023). Copper-based nanoparticles against microbial infections. Wiley Interdiscip. Rev. Nanomed. Nanobiotechnol..

[14] Pramanik A., Laha D., Bhattacharya D., Pramanik P., Karmakar P. (2012). A novel study of antibacterial activity of copper iodide nanoparticle mediated by DNA and membrane damage. Colloids Surf. B Biointerfaces.

[15] McDonnell G., Russell A.D. (1999). Antiseptics and disinfectants: Activity, action, and resistance. Clin. Microbiol. Rev..

[16] Pesticide Research Institute Iodine: Technical Evaluation Report; Compiled for the USDA National Organic Program, 2 January 2015. https://www.ams.usda.gov/sites/default/files/media/Iodine%20TR%202015.pdf.

[17] Awed A.S., El-Sayyad G.S., El-Ghandour A., Hameed M.F.O., Abdel Maksoud M.I.A., El-Batal A.I., Obayya S.S.A. (2021). Unveiling Antimicrobial Activity of Metal Iodide (CuI, AgI, and PbI_2_) Nanoparticles: Towards Biomedical Surfaces Applications. J. Clust. Sci..

[18] Hemmat M.A., Asghari S., Bakhshesh M., Mahmoudifard M. (2023). Copper iodide decorated graphene oxide as a highly efficient antibacterial and antiviral nanocomposite. Inorg. Chem. Commun..

[19] Archana K.M., Rajagopal R., Krishnaswamy V.G., Aishwarya S. (2021). Application of green synthesised copper iodide particles on cotton fabric-protective face mask material against COVID-19 pandemic. J. Mater. Res. Technol..

[20] Phetcharat P., Sangsanoh P., Choipang C., Chaiarwut S., Suwantong O., Chuysinuan P., Supaphol P. (2023). Curative effects of copper iodide embedded on gallic acid incorporated in a poly(vinyl alcohol) liquid bandage. Gels.

[21] Takeda Y., Jamsransuren D., Nagao T., Fukui Y., Matsuda S., Ogawa H. (2021). Application of copper iodide nanoparticle-doped film and fabric to in-activate SARS-CoV-2 via the virucidal activity of cuprous ions (Cu^+^). Appl. Environ. Microbiol..

[22] Chen A.X., Beins D.K.R., Wang Y., Luo H.K., Yang Y.Y., Li N. (2024). Fast-Acting and Skin-Compatible Antimicrobial Coating on Cotton Fabrics via In Situ Self-Assembly of Phosphine-Coordinated Copper Iodide Clusters. ACS Appl. Eng. Mater..

[23] Singh H., Desimone M.F., Pandya S., Jasani S., George N., Adnan M., Aldarhami A., Bazaid A.S., Alderhami S. (2023). Revisiting the green synthesis of nanoparticles: Uncovering influences of plant extracts as reducing agents for enhanced synthesis efficiency and its biomedical applications. Int. J. Nanomed..

[24] Tang Q., Qian Y., Yang D., Qiu X., Qin Y., Zhou M. (2020). Lignin-based nanoparticles: A review on their preparations and applications. Polymers.

[25] Murgueitio Herrera E., Jacome G., Stael C., Arroyo G., Izquierdo A., Debut A., Delgado P., Montalvo G. (2024). Green Synthesis of Metal Nanoparticles with Borojó (Borojoa patinoi) Extracts and Their Application in As Removal in Water Matrix. Nanomaterials.

[26] Simirgiotis M.J., Schmeda-Hirschmann G., Bórquez J., Kennelly E.J. (2013). The *Passiflora tripartita* (Banana Passion) fruit: A source of bioactive flavonoid C-glycosides isolated by HSCCC and characterized by HPLC–DAD–ESI/MS/MS. Molecules.

[27] Romaní D.R., Cotos R.C., Suca F. (2021). Aprovechamiento de los residuos del fruto de *Passiflora tripartita*. Sci. Agropecu..

[28] He X., Luan F., Yang Y., Wang Z., Zhao Z., Fang J., Wang M., Zuo M., Li Y. (2020). *Passiflora edulis*: An insight into current researches on phytochemistry and pharmacology. Front. Pharmacol..

[29] Pereira Z.C., Cruz J.M.d.A., Corrêa R.F., Sanches E.A., Campelo P.H., Bezerra J.d.A. (2023). Passion fruit (*Passiflora* spp.) pulp: A review on bioactive properties, health benefits and technological potential. Food Res. Int..

[30] Ramli A.N.M., Manap N.W.A., Bhuyar P., Azelee N.I.W. (2020). Passion fruit (*Passiflora edulis*) peel powder extract and its application towards antibacterial and antioxidant activity on the preserved meat products. SN Appl. Sci..

[31] Pelegrini P.B., Noronha E.F., Muniz M.A.R., Vasconcelos I.M., Chiarello M.D., Oliveira J.T.A., Franco O.L. (2006). An antifungal peptide from passion fruit (*Passiflora edulis*) seeds with similarities to 2S albumin proteins. Biochim. Biophys. Acta Proteins Proteom..

[32] Laha D., Bhattacharya D., Pramanik A., Santra C.R., Pramanik P., Karmakar P. (2012). Evaluation of copper iodide and copper phosphate nanoparticles for their potential cytotoxic effect. Toxicol. Res..

[33] Awashra M., Młynarz P. (2023). The toxicity of nanoparticles and their interaction with cells: An in vitro metabolomic perspective. Nanoscale Adv..

[34] Xiong P., Huang X., Ye N., Lu Q., Zhang G., Peng S., Wang H., Liu Y. (2022). Cytotoxicity of metal-based nanoparticles: From mechanisms and methods of evaluation to pathological manifestations. Adv. Sci..

[35] Gupta G., Cappellini F., Farcal L., Gornati R., Bernardini G., Fadeel B. (2022). Copper oxide nanoparticles trigger macrophage cell death with misfolding of Cu/Zn superoxide dismutase 1 (SOD1). Part. Fibre Toxicol..

[36] Saito Y., Ando M., Omura G., Yasuhara K., Yoshida M., Takahashi W., Yamasoba T. (2017). Copper oxide nanoparticles induce apoptosis in cancer cells but not in normal cells through oxidative stress-mediated pathways. Int. J. Nanomed..

[37] Basu S., Das D., Morgan D., Hazra B., Saha A., Sen K. (2023). Green synthesis of copper iodide nanoparticles: Gamma irradiation for spectroscopic sensing of cancer biomarker CA 19-9. J. Radioanal. Nucl. Chem..

[38] Darnige P., Thimont Y., Presmanes L., Barnabé A. (2023). Insights into stability, transport, and thermoelectric properties of transparent p-type copper iodide thin films. J. Mater. Chem. C.

[39] Byranvand M.M., Kharat A.N. (2014). Triangular-like cuprous iodide nanostructures: Green and rapid synthesis using sugar beet juice. Rom. J. Biochem..

[40] Ghazal N., Madkour M., Nazeer A.A., Obayya S.S.A., Mohamed S.A. (2021). Electrochemical capacitive performance of thermally evaporated Al-doped CuI thin films. RSC Adv..

[41] Sielska A., Skuza L. (2025). Copper Nanoparticles in Aquatic Environment: Release Routes and Oxidative Stress-Mediated Mechanisms of Toxicity to Fish in Various Life Stages and Future Risks. Curr. Issues Mol. Biol..

[42] Liu N., Tong L., Li K., Dong Q., Jing J. (2024). Copper-nanoparticle-induced neurotoxic effect and oxidative stress in the early developmental stage of zebrafish (*Danio rerio*). Molecules.

[43] Menges F. (2022). SpectraGryph—Optical Spectroscopy Software.

[44] Javaid R., Anwar H., Jamil Y., Abbas B., Iqbal M., Ahmed S., Islam A., Batool F., Khalid M., Elahi U. (2020). Study of the morphological transformation of copper iodide nanostructures prepared by sugar beet mediated route. J. Ovonic Res..

[45] Lim J., Yeap S.P., Che H.X., Low S.C. (2013). Characterization of magnetic nanoparticle by dynamic light scattering. Nanoscale Res. Lett..

[46] Schindelin J., Arganda-Carreras I., Frise E., Kaynig V., Longair M., Pietzsch T., Preibisch S., Rueden C., Saalfeld S., Schmid B. (2012). Fiji: An open-source platform for biological-image analysis. Nat. Methods.

[47] Bruker XFlash^®^ 6|30 Detector. https://www.bruker.com/en/products-and-solutions/elemental-analyzers/eds-wds-ebsd-SEM-Micro-XRF/detectors/xflash-6-30-detector.html.

[48] TESCAN ORSAY HOLDING, a.s. TESCAN MIRA: High-Resolution FEG SEM for Research, Quality Control, and Failure Analysis. https://tescan.com/product-portfolio/sem/mira.

[49] Ted Pella, Inc. Scanning Electron Microscopy Calibration. https://www.tedpella.com/calibration_html/SEM_Calibration.aspx.

[50] Willis R., Conner T. (2003). Guidelines for the Application of SEM/EDX Analytical Techniques for Fine and Coarse PM Samples.

[51] Brown J., Chen C., Fernández M., Carr D. (2023). Urban endoliths: Incidental microbial communities occurring inside concrete. AIMS Microbiol..

[52] Verdier T., Coutand M., Bertron A., Roques C. (2014). A review of indoor microbial growth across building materials and sampling and analysis methods. Build. Environ..

[53] Wilson K. (2001). Preparation of genomic DNA from bacteria. Curr. Protoc. Mol. Biol..

[54] Tamura K., Stecher G., Kumar S. (2021). MEGA11: Molecular Evolutionary Genetics Analysis Version 11. Mol. Biol. Evol..

[55] Olmedo Fonseca P.C. (2019). Evaluación del Potencial Antifúngico de los Extractos Crudos de Biosurfactante Producidos por *Bacillus* spp. Aislados de Diferentes Fuentes Ambientales del Ecuador, a Nivel de Laboratorio. Bachelor’s Thesis.

[56] Balouiri M., Sadiki M., Ibnsouda S.K. (2016). Methods for in vitro evaluating antimicrobial activity: A review. J. Pharm. Anal..

[57] Ghasemi M., Turnbull T., Sebastian S., Kempson I. (2021). The MTT assay: Utility, limitations, pitfalls, and interpretation in bulk and single-cell analysis. Int. J. Mol. Sci..

[58] Riss T.L., Moravec R.A., Niles A.L., Duellman S., Benink H.A., Worzella T.J., Minor L. (2016). Cell Viability Assays. Assay Guidance Manual.

[59] Mosmann T. (1983). Rapid colorimetric assay for cellular growth and survival: Application to proliferation and cytotoxicity assays. J. Immunol. Methods.

[60] Denizot F., Lang R. (1986). Rapid colorimetric assay for cell growth and survival: Modifications to the tetrazolium dye procedure giving improved sensitivity and reliability. J. Immunol. Methods.

[61] Nelissen I., Haase A., Anguissola S., Rocks L., Jacobs A., Willems H., Riebeling C., Luch A., Piret J.P., Toussaint O. (2020). Improving quality in nanoparticle-induced cytotoxicity testing by a tiered inter-laboratory comparison study. Nanomaterials.

[62] Ong K.J., MacCormack T.J., Clark R.J., Ede J.D., Ortega V.A., Felix L.C., Dang M.K.M., Ma G., Fenniri H., Veinot J.G.C. (2014). Widespread nanoparticle-assay interference: Implications for nanotoxicity testing. PLoS ONE.

[63] (2009). Biological Evaluation of Medical Devices—Part 5: Tests for In Vitro Cytotoxicity.

[64] Gruber S., Nickel A. (2023). Toxic or not toxic? The specifications of the standard ISO 10993-5 are not explicit enough to yield comparable results in the cytotoxicity assessment of an identical medical device. Front. Med. Technol..

[65] (2020). Standard Test Method for Obtaining and Testing Drilled Cores and Sawed Beams of Concrete.

[66] Ferguson Structural Engineering Laboratory (2016). Procedure for Compression Testing of Concrete Cylinders and Cores (Rev. 0).

[67] (2023). Standard Test Method for Compressive Strength of Cylindrical Concrete Specimens.

[68] (2023). Standard Practice for Use of Unbonded Caps in Determination of Compressive Strength of Hardened Cylindrical Concrete Specimens.

[69] Khoury S., Aliabdo A.A.-H., Ghazy A. (2014). Reliability of core test–Critical assessment and proposed new approach. Alex. Eng. J..

[70] Ju M., Park K., Oh H. (2017). Estimation of compressive strength of high strength concrete using non-destructive technique and concrete core strength. Appl. Sci..

[71] Vasco C., Ruales J., Kamal-Eldin A. (2008). Total phenolic compounds and antioxidant capacities of major fruits from Ecuador. Food Chem..

[72] Hider R.C., Liu Z.D., Khodr H.H. (2001). Metal chelation of polyphenols. Methods Enzymol..

[73] Kozioł A., Środa-Pomianek K., Górniak A., Wikiera A., Cyprych K., Malik M. (2022). Structural determination of pectins by spectroscopy methods. Coatings.

[74] Pasieczna-Patkowska S., Cichy M., Flieger J. (2025). Application of Fourier transform infrared (FTIR) spectroscopy in characterization of green synthesized nanoparticles. Molecules.

[75] Qiu X., Ou Y., Lu S., Liang Y., Zhang Y., Li M., Li G., Ma H., Wu Y., He Z. (2024). Study of the structure and bioactivity of polysaccharides from different parts of *Stemona tuberosa* Lour. Molecules.

[76] Wongsa P., Phatikulrungsun P., Prathumthong S. (2022). FT-IR characteristics, phenolic profiles and inhibitory potential against digestive enzymes of 25 herbal infusions. Sci. Rep..

[77] Scimeca M., Bischetti S., Lamsira H.K., Bonfiglio R., Bonanno E. (2018). Energy Dispersive X-ray (EDX) microanalysis: A powerful tool in biomedical research and diagnosis. Eur. J. Histochem..

[78] Fan W.W., Gualtieri A.F., Hamilton A., Patel J.P., Salmond J.A. (2025). Determining factors affecting the accuracy of SEM-EDX data-based quantitative chemical analysis for identifying naturally occurring individual carcinogenic erionite fibers. Sci. Rep..

[79] Pereira M.G., Maciel G.M., Haminiuk C.W.I., Bach F., Hamerski F., Scheer A.d.P., Corazza M.L. (2019). Effect of extraction process on composition, antioxidant and antibacterial activity of oil from yellow passion fruit (*Passiflora edulis Var. Flavicarpa*) seeds. Waste Biomass Valorization.

[80] Zakharova O.V., Godymchuk A.Y., Gusev A.A., Gulchenko S.I., Vasyukova I.A., Kuznetsov D.V. (2015). Considerable variation of antibacterial activity of Cu nanoparticles suspensions depending on the storage time, dispersive medium, and particle sizes. BioMed Res. Int..

[81] Fernandez A.C., Rajagopal R. (2020). Green synthesis, characterization, catalytic and antibacterial studies of copper iodide nanoparticles synthesized using *Brassica oleracea var. capitata f. rubra* extract. Chem. Data Collect..

[82] Chen A.X., Lau H.Y., Teo J.Y., Wang Y., Choong D.Z.Y., Wang Y., Luo H.K., Yang Y.Y., Li N. (2023). Water-mediated in situ fabrication of CuI nanoparticles on flexible cotton fabrics as a sustainable and skin-compatible coating with broad-spectrum antimicrobial efficacy. ACS Appl. Nano Mater..

